# Treatment with a Combination of Metformin and 2-Deoxyglucose Upregulates Thrombospondin-1 in Microvascular Endothelial Cells: Implications in Anti-Angiogenic Cancer Therapy

**DOI:** 10.3390/cancers11111737

**Published:** 2019-11-06

**Authors:** Samson Mathews Samuel, Noothan Jyothi Satheesh, Suparna Ghosh, Dietrich Büsselberg, Yasser Majeed, Hong Ding, Chris R. Triggle

**Affiliations:** 1 Department of Pharmacology, Weill Cornell Medicine-Qatar, Education City, Qatar Foundation, Doha 24144, Qatar; noothanjyothi@yahoo.co.in (N.J.S.); gsuparna14@gmail.com (S.G.); yam2013@qatar-med.cornell.edu (Y.M.); hod2005@qatar-med.cornell.edu (H.D.); 2 Department of Physiology and Biophysics, Weill Cornell Medicine-Qatar, Education City, Qatar Foundation, Doha 24144, Qatar; dib2015@qatar-med.cornell.edu; 3 Department of Medical Education, Weill Cornell Medicine-Qatar, Education City, Qatar Foundation, Doha 24144, Qatar

**Keywords:** angiogenesis, cancer, combination therapy, metformin, organic cation transporter (OCT), tumor endothelial cells

## Abstract

Metformin, the most widely used anti-diabetic drug, also exhibits anti-cancer properties; however, the true potential of metformin as an anticancer drug remains largely unknown. In this study using mouse microvascular endothelial cells (MMECs), we investigated the effects of metformin alone or in combination with the glycolytic inhibitor, 2-deoxyglucose (2DG), on angiogenesis-a process known to be an integral part of tumor growth, cancer cell survival and metastasis. MMECs were exposed to 2DG (1–10 mM) for 48 h in the absence or presence of metformin (2 mM). The status of angiogenic and anti-angiogenic marker proteins, proteins of the mTOR pathway and cell-cycle-related proteins were quantified by Western blot analysis. Assays for cell proliferation, migration and tubulogenesis were also performed. We observed robust up-regulation of anti-angiogenic thrombospondin-1 (TSP1) and increased TSP1-CD36 co-localization with a marked decrease in the levels of phosphorylated vascular endothelial growth factor receptor-2 (pVEGFR2; Y1175) in 2DG (5 mM) exposed cells treated with metformin (2 mM). Additionally, treatment with metformin and 2DG (5 mM) inhibited the Akt/mTOR pathway and down-regulated the cell-cycle-related proteins such as p-cyclin B1 (S147) and cyclins D1 and D2 when compared to cells that were treated with either 2DG or metformin alone. Treatment with a combination of 2DG (5 mM) and metformin (2 mM) also significantly decreased cell proliferation, migration and tubulogenic capacity when compared to cells that were treated with either 2DG or metformin alone. The up-regulation of TSP1, inhibition of cell proliferation, migration and tubulogenesis provides support to the argument that the combination of metformin and 2DG may prove to be an appropriate anti-proliferative and anti-angiogenic therapeutic strategy for the treatment of some cancers.

## 1. Introduction

For the majority of patients with type 2 diabetes, metformin (1,1-dimethylbiguanide hydrochloride), is the drug prescribed as, in addition to its efficacy as an anti-hyperglycemic drug, it has proven cardiovascular protective actions and, for most patients, minimal side effects [1]. In addition to its anti-diabetic actions it has also been reported that patients treated with metformin have a reduced risk of cancer [1,2,3,4,5]. For instance, type 2 diabetic patients treated with metformin have a reduced risk of cancer such as colorectal, prostate and breast cancer [2]. Despite contradictory results from randomized trials, observational studies have reported a reduced risk of pancreatic cancer in patients treated with metformin [6,7]. Thus, it is possible that the ability and mechanism of action whereby metformin reduces the risk of cancer will not only differ between types of cancer, but also patient characteristics [6].

As a result of this increasing interest in determining the molecular mechanism of the putative anti-cancer effects of metformin data from several in vitro studies using cancer cell lines support the hypothesis that metformin can induce apoptosis and inhibit cancer cell growth [8,9]. However, in many studies, such anti-cancer effects of metformin were only observed at very high concentrations (>5 mM) [8,9]. Following the oral administration of metformin for the treatment of type 2 diabetes, the concentration of metformin in the hepatic circulation may reach 50 μM; however, the peak plasma concentration of metformin is no higher than 20 μM and trough levels <5 μM [8,9,10]. At physiological pH, metformin exists as a cation (>99.9%) and to what, if any, extent metformin accumulates in cells depends on the relative expression of the inward and outward organic cation transporters-1, 2 and 3 (OCT1/2/3), plasma membrane monoamine trasporter (PMAT) and multidrug and toxin extrusion protein-1 and 2 (MATE1/2), that regulate the absorption and extrusion of metformin [11]. In an in vivo model of breast cancer, metformin accumulation and subsequent tumor regression was reported to correlate with expression of OCT2, while in BT-20 breast cancer cells, metformin uptake was enhanced and associated with an overexpression of OCT3 [12,13]. Therefore, a selective transport of metformin into cancer cells would facilitate the accumulation of millimolar levels of metformin within the target cancer cells [8,9,10]. It is also possible that the anti-cancer effects of metformin may result from improvement in insulin sensitivity and be secondary to its anti-hyperglycemic effect [9,14]. In conjunction with reports indicating the reduced efficacy of the anti-cancer/anti-proliferative effect of metformin in the presence of glucose [15], we have shown that mM concentrations of metformin (2 mM), but not μM (50 μM) have a differential effect in mouse microvascular endothelial cells (MMECs), depending on the presence (11 mM) or absence (glucose-starved) of glucose in the culture system [16,17]. While 50 μM metformin was ineffective, treatment with 2 mM metformin significantly reversed glucose starvation-induced autophagy (a pro-survival mechanism in cancer cells) [16,18,19]. In addition, we observed cell cycle arrest in the gap phase-2/mitosis (G2/M) phase of the cell cycle, a decrease in cell viability and inhibition of the protein kinase B/mammalian target of rapamycin (Akt/mTOR) pathway, while the levels of apoptotic cleaved/activated caspase 3 (Casp3) increased, leading to cell death in glucose-starved MMECs treated with metformin (2 mM) [16]. In agreement with previous reports [20], we have also shown: (1) reversal of glucose starvation induced pro-survival autophagy in MDA-MB 231 triple-negative breast cancer cells treated with metformin (2 mM) and (2) reversal of 2-deoxyglucose (2DG) induced pro-survival autophagy in metformin-treated (2 mM) MMECs, triple-negative breasts cancer cells (MDA-MB 231) and human umbilical vein endothelial cells (HUVECs) [16]. Thus, the effects of millimolar concentrations of metformin on autophagy are not limited to endothelial cells.

The potential limitation in studying the effect of metformin in different forms of cancer is that the optimal dose of metformin that demonstrates efficacy in the treatment of cancer might vary subject to, for instance, the signaling pathway whereby metformin mediates its anti-cancer actions, as well as differences in the sensitivity of the cancer to metformin. In such a scenario, identifying a common target whereby an optimal dose of metformin could be used as a therapeutic strategy should prove beneficial in the treatment of various forms of cancer. Angiogenesis, a key function of endothelial cells (ECs), forms an integral part of tumor growth, cancer cell survival and metastasis in different forms of cancer [21]. Targeting tumor angiogenesis to inhibit vascularization of growing tumors [22] is a key therapeutic approach for the treatment of many different kinds of cancer. In comparison with normal ECs, tumor ECs (TECs) showcase an altered metabolism and exhibit a defective endothelial monolayer that causes abnormal sprouting, leading to the formation of leaky blood vessels that support tumor progression and metastasis [16,23,24,25]. Additionally, angiogenesis in a growing tumor also results from the robust upregulation of the synthesis and constant release of angiogenic growth factors such as vascular endothelial growth factor (VEGF) as a response to hypoxia/ischemia in the tumor microenvironment [26]. Furthermore, while the glycolytic flux is doubled in TECs, there is a decrease in the rate of oxidative phosphorylation, thereby exhibiting characteristics of the Warburg effect, a prominent feature of cancer cells [27]. Interestingly, metformin reportedly increases the levels of anti-angiogenic thrombospondin-1 (TSP1) in the serum of women with polycystic ovarian syndrome and thereby inhibited angiogenesis [28].

We hypothesized that using metformin in combination with a glycolytic inhibitor, such as 2DG, to target the altered metabolism specifically associated with TECs would have a synergistic effect, thereby inhibiting tumor angiogenesis and, ultimately, curbing tumor growth. The concept of using metformin and 2DG, individually and in combination, has gained importance as a potential anti-cancer therapeutic strategy. However, the effects of a combination of metformin and 2DG on angiogenesis in TECs and cell fate have not been previously investigated. In the current study, we have examined the effect of metformin in combination with 2DG on VEGF overexpressing MMECs that can form well-differentiated angiosarcomas in mice [29,30,31]. In this regard, the levels of angiogenic and anti-angiogenic markers, levels of proteins of the Akt/mTOR pathway and cell-cycle-related proteins and the rate of cell proliferation and migration were determined.

## 2. Results

### 2.1. Metformin (2 mM) Increases the Levels of TSP1 in Glucose-Starved MMECs

**Figure 1:**
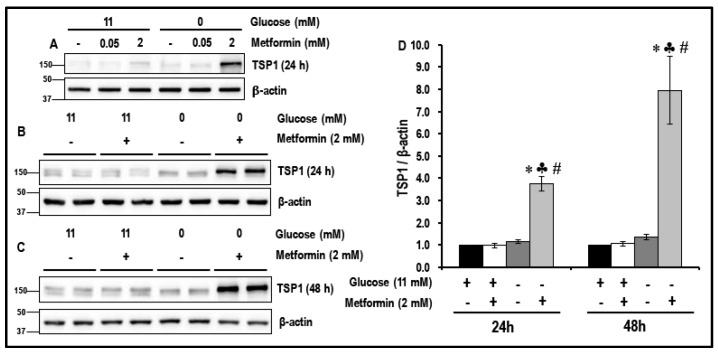
The levels of anti-angiogenic thrombospondin-1 (TSP1) in metformin-treated normal glucose-exposed and glucose-starved mouse microvascular endothelial cells (MMECs) (24 h and 48 h): Western blot images, (**A**) show the effect of 50 μM or 2 mM metformin on the levels of TSP1 in normal glucose (11 mM)-exposed and glucose-starved cells, (**B**) and (**C**) show the effect of 2 mM metformin on the levels of TSP1 in normal glucose (11 mM)-exposed and glucose-starved cells after 24 h and 48 h in culture, respectively. Bar graphs (**D**) represent the levels (normalized with b-actin loading controls) of TSP1 in the cells after 24 h and 48 h in culture. * *p* < 0.05 indicates significance when compared to non-treated controls (11 mM glucose-exposed MMECs), ♣ *p* < 0.05 indicates significance when compared to 11 mM glucose + 2 mM metformin-treated cells and # *p* < 0.05 indicates significance when compared to glucose-starved cells.

We have previously reported the, reversal of glucose-starvation-induced pro-survival autophagy, inhibition of the Akt/mTOR pathway, activation of caspase 3, G2/M cell cycle arrest and increased cell death in metformin (2 mM)-treated glucose-starved MMECs [16]. In the current study, we determined the levels of anti-angiogenic TSP1 in a similar experimental protocol of glucose starvation as previously described [16]. MMECs were glucose-starved in the absence or presence of metformin (50 μM or 2 mM) for 24 h and 48 h. Normal glucose (11 mM) exposed cells maintained in the absence or presence of metformin were considered as normal controls. The levels of TSP1 in MMECs were not altered with glucose starvation alone when compared to normal glucose-exposed cells. However, we observed a significant increase in the levels of anti-angiogenic TSP1 after 24 h (~3.9-fold; Figure 1A,B,D; ♣ *p* < 0.05) and 48 h (~7.6-fold, Figure 1C,D, ♣ *p* < 0.05) in glucose-starved MMECs exposed to 2 mM metformin when compared to the metformin-treated normal glucose (11 mM)-exposed cells.

In comparison, exposure to 50 μM metformin (50 μM is the putative upper level of metformin in the blood to the liver when used as an oral anti-hyperglycemic agent) did not result in any change in the levels of TSP1 in either normal glucose-exposed cells or glucose-starved cells (Figure 1A).

### 2.2. Treatment with a Combination of 2DG and Metformin Up-Regulates Expression of Anti-Angiogenic TSP-1 in MMECs

The levels of TSP1 significantly increased in metformin (2 mM)-treated glucose-starved MMECs. It is, however, impossible to starve cells of glucose in a clinical setting. We, therefore, hypothesized that in a clinical setting, using metformin in a microenvironment that mimics glucose starvation, such as glycolytic inhibition using inhibitors, should have a similar effect on the levels of TSP1, as observed in metformin-treated glucose-starved MMECs. To test this hypothesis, MMECs were exposed to varying concentrations of 2DG (1 mM, 2 mM, 5 mM, 7.5 mM and 10 mM) in the absence or presence of metformin (2 mM) for 48 h, as described in Section 4.3 under ‘cell treatments’.

**Figure 2:**
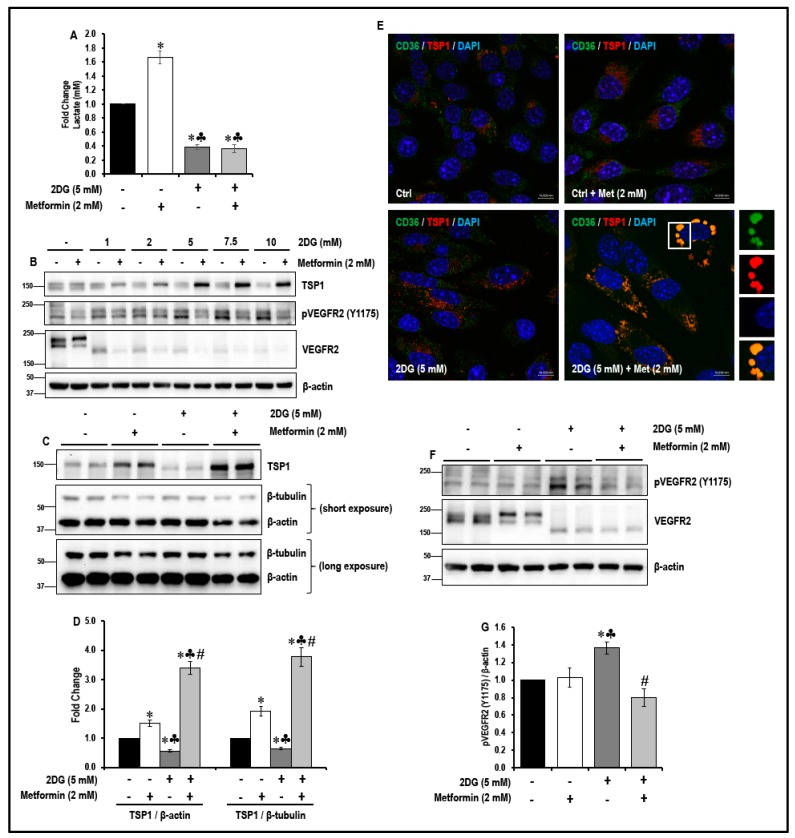
Effect of metformin treatment on glycolysis, levels of TSP1, TSP1-platelet glycoprotein IV/scavenger receptor class B member 3 (CD36) co-localization and levels of phosphorylated vascular endothelial growth factor receptor-2 (pVEGFR2; Y1175) in normal glucose and 2-deoxyglucose (2DG)-exposed MMECs (48 h): Bar graphs (**A**) represent fold change in the levels of lactate (mM) in the cells treated with 2DG (5 mM) with or without metformin (2 mM), after 48 h in culture. Representative Western blots (**B**) show the levels of TSP1, pVEGFR2 (Y1175) and VEGFR2 in MMECs treated with varying concentrations of 2DG with or without metformin (2 mM), after 48 h in culture. Representative Western blots (**C**) show the levels of TSP1 while (**F**) show the levels of pVEGFR2 (Y1175) and VEGFR2 in cells exposed to 2DG (5 mM) in the presence or absence of metformin (2 mM), after 48 h in culture. Non-treated cells and cells treated with metformin (2 mM) alone were used as suitable controls. Bar graphs (**D**) represent levels of TSP1/β-actin and TSP1/β-tubulin (normalized against β-actin and β-tubulin loading controls) while bar graphs (**G**) represent ratio of pVEGFR2 (Y1175)/β-actin in the cells treated with 2DG (5 mM) with or without metformin (2 mM), after 48 h in culture. * *p* < 0.05 indicates significance when compared to non-treated controls, ♣ *p* < 0.05 indicates significance when compared to control + 2 mM metformin-treated cells and # *p* < 0.05 indicates significance when compared to 2DG (5 mM)-treated cells. Representative immunofluorescence confocal images (**E**; from three experiments) show TSP1 (red) and CD36 (green) staining in MMECs exposed to 2DG (5 mM) in the presence or absence of metformin (2 mM). Nuclear stain, 4′,6-diamidino-2-phenylindole (DAPI; blue), has been used to stain the nuclei. Scale bar = 10 μm.

We first verified whether 2DG (5 mM) inhibited glycolysis in MMECs. 2DG (5 mM) treatment in metformin (2 mM)-exposed and non-treated MMECs significantly reduced glycolysis by ~2.7-fold and ~2.6-fold, respectively, when compared to non-treated controls (Figure 2A; * *p* < 0.05). Interestingly, metformin (2 mM) treatment alone significantly increased (~1.7-fold; * *p* < 0.05) glycolysis in MMECs when compared to non-treated controls (Figure 2A).

A marked decrease in the levels of TSP1 was observed in MMECs treated with 2DG alone. We observed a significant decrease (~1.8-fold; Figure 2B,C,D; * *p* < 0.05) in TSP1 levels in 2DG (5 mM)-treated controls when compared to non-treated controls. Treatment of MMECs with a combination of 2DG (2–10 mM) and metformin (2 mM) for 48 h markedly increased the levels of TSP1 (Figure 2B) when compared to the MMECs treated with either 2DG or metformin (2 mM) alone. We observed that TSP1 levels peaked (Figure 2B) when using 2 mM metformin and 5 mM 2DG in combination and, therefore, final comparisons for statistical analysis and graphical representations were made between the cells treated with the 2DG (5 mM) + metformin (2 mM) combination and the cells treated with either 2DG (5 mM) or metformin (2 mM) alone. We observed that after 48 h in culture, in MMECs treated with a combination of 2DG (5 mM) and metformin (2 mM), there was a significant increase in the levels of TSP1 (~2.8-fold and 6.4-fold; Figure 2B,CD; ♣ *p* < 0.05 and # *p* < 0.05), respectively, when compared to the MMECs treated with either metformin (2 mM) or 2DG (5 mM) alone.

### 2.3. Treatment with a Combination of 2DG and Metformin Increases TSP1-Platelet Integral Membrane Glycoprotein IV/Scavenger Receptor Class B Member-3 (CD36) Co-Localization and Decreases VEGFR2 Phosphorylation in MMECs

In microvascular endothelial cells, CD36 (a multi-ligand scavenger receptor) acts as a receptor for TSP1 [32]. In our current study, immunofluorescence double-antibody staining for TSP1 (red) and CD36 (green), followed by confocal microscopy, showed a marked increase in the co-localization of TSP1 and CD36 (yellow) (Figure 2E) in MMECs treated with a combination of 2DG (5 mM) + metformin (2 mM) when compared to the MMECs treated with either metformin (2 mM) or 2DG (5 mM) alone.

Since we observed a significant increase in the levels of TSP1 (Figure 2B–D) and TSP1-CD36 association (Figure 2E) in MMECs treated with a combination of 2DG and metformin, we then studied the effect of this combination on the phosphorylation and activation of the VEGFR2 receptor, which is a key molecule responsible for the activation of the angiogenic cascade in endothelial cells. While metformin treatment alone did not affect the total VEGFR2 levels in MMECs, treatment with 2DG alone markedly decreased the levels of total VEGFR2. The levels of total VEGFR2 were further decreased in the cells treated with a combination of 2DG and metformin (Figure 2B,F). Thus, MMECs treated with 2DG alone showed a significant increase in the levels of pVEGFR2 (Y1175; ~1.4-fold; # *p* < 0.05, Figure 2B,F,G) when compared to non-treated controls. However, there was a significant decrease in the levels of pVEGFR2 (Y1175; Figure 2B,F,G) in MMECs treated with a combination of 2DG (5 mM) and metformin (2 mM) when compared to the MMECs treated with either metformin (2 mM) or 2DG alone. We observed a significant decrease in the levels of pVEGFR2 (Y1175; ~1.7-fold; # *p* < 0.05, Figure 2B,F,G) in MMECs treated with a combination of 2DG (5 mM) and metformin (2 mM) when compared to the MMECs treated with 2DG (5 mM) alone.

### 2.4. Treatment with a Combination of 2DG and Metformin Inhibits the Akt/mTOR Pathway in MMECs

**Figure 3:**
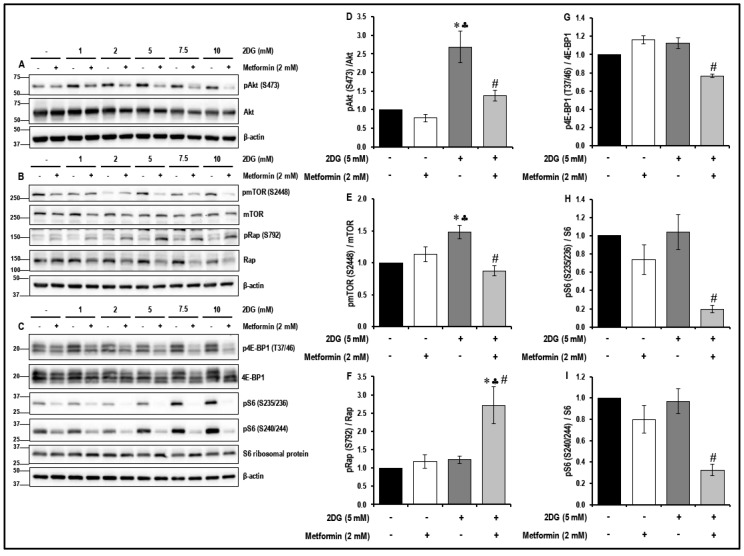
The levels of protein kinase B/mammalian target of rapamycin (Akt/mTOR) pathway-related proteins in metformin-treated normal glucose- and 2DG-exposed MMECs (48 h): Representative Western blot images (**A**) show the levels of phosphorylated Akt (S473) and Akt, (**B**) show the levels of phosphorylated mTOR (S2448), mTOR, phosphorylated Raptor-pRap (S792) and Raptor (Rap) and (**C**) show the levels of phosphorylated eukaryotic translation initiation factor 4E (eIF4E)-binding protein 1-p4E-BP1 (T37/46), 4E-BP1, phosphorylated-S6 ribosomal protein-pS6 (S235/236), pS6 (S240/244), S6 ribosomal protein after 48 h in culture. Non-treated cells and cells treated with metformin (2 mM) alone were used as suitable controls. Bar graphs represent the ratio of (normalized against β-actin loading controls) (**D**) pAkt (S473)/Akt, (**E**) pmTOR (S2448)/mTOR, (**F**) pRap (S792)/Rap, (**G**) p4E-BP1 (T37/46)/4E-BP1, (**H**) pS6 (S235/236)/S6 ribosomal protein and (**I**) pS6 (S240/244)/S6 ribosomal protein after 48 h in culture. * *p* < 0.05 indicates significance when compared to non-treated controls, ♣ *p* < 0.05 indicates significance when compared to control + 2 mM metformin-treated cells and # *p* < 0.05 indicates significance when compared to 2DG (5 mM)-treated cells.

It has been reported that dysregulation of the Akt/mTOR pathway plays a key role in the initiation and progression of different forms of cancer [16] and, therefore, we studied the effects of treatment with 2DG, metformin and their combination on the Akt/mTOR pathway in MMECs (Figure 3).

Treatment of MMECs with metformin (2 mM) alone caused a marked reduction in the levels of phosphorylated-S6 ribosomal protein - pS6 (S235/236) (Figure 3C,H) and pS6 (S240/244) (Figure 3C,I) in the cells after 48 h in culture when compared to the non-treated control cells. While treatment with 2DG (5 mM) alone for 48 h significantly increased the levels of phosphorylated Akt - pAkt (S473) (~2.7-fold; * *p* < 0.05, Figure 3A,D) and phosphorylated mTOR-pmTOR (S2448) (~1.5-fold, * *p* < 0.05, Figure 3B,E) in the MMECs when compared to the non-treated cells, it did not significantly alter the levels of the downstream targets of mTOR. However, treatment of MMECs with a combination of 2DG (5 mM) and metformin (2 mM) for 48 h had a profound effect to inhibit the mTOR pathway, as evidenced by a significant increase in the levels of phosphorylated-Raptor-pRap (S792) (~2.3-fold and 2.2-fold, ♣ *p* < 0.05 and # *p* < 0.05, Figure 3B,F) and decrease in the levels of pAkt (S473) (~1.8-fold and 2-fold, ♣ *p* < 0.05 and # *p* < 0.05, Figure 3A,D), pmTOR (S2448) (~1.3-fold and 1.7-fold, ♣ *p* < 0.05 and # *p* < 0.05, Figure 3B,E), phosphorylated eukaryotic translation initiation factor 4E (eIF4E)-binding protein 1, p4E-BP1 (T37/46) (~1.5-fold and 1.5-fold, ♣ *p* < 0.05 and # *p* < 0.05, Figure 3C,G), pS6 (S235/236) (~3.8-fold and 5.4-fold, ♣ *p* < 0.05 and # *p* < 0.05, Figure 3C,H) and pS6 (S240/244) (~2.5-fold and 3-fold, ♣ *p* < 0.05 and # *p* < 0.05, Figure 3C,I), respectively, when compared to cells treated with either metformin (2 mM) or 2DG (5 mM) alone.

### 2.5. Treatment with a Combination of 2DG and Metformin Inhibits the Cell-Cycle-Related Proteins in MMECs

**Figure 4:**
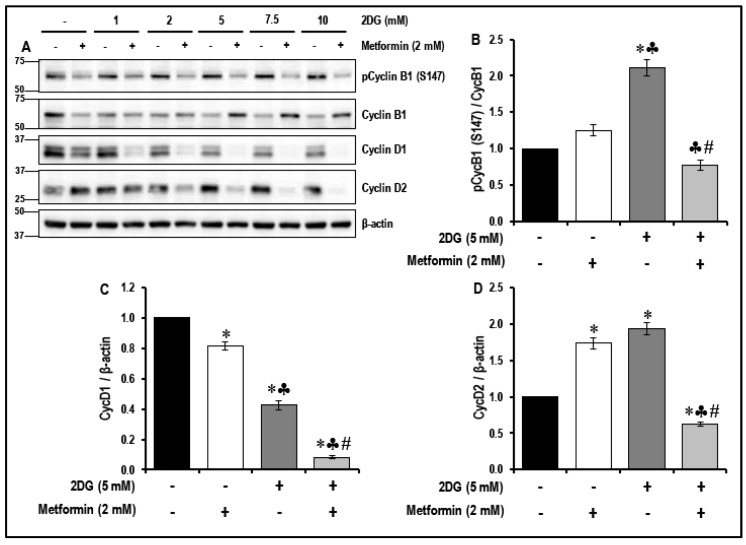
The levels of phosphorylated cyclin B1 (pCycB1; S147), cyclin D1 (CycD1) and cyclin D2 (CycD2) in metformin treated normal glucose- and 2DG-exposed MMECs (48 h): Representative Western blots (**A**) shows the levels of pCycB1 (S147), CycB1, Cyc D1 and D2 in cells treated with varying concentrations of 2DG in the presence or absence of metformin (2 mM), after 48 h in culture. Non-treated cells and cells treated with metformin (2 mM) alone were used as suitable controls. Bar graphs represent the ratio of (normalized against β-actin loading controls) pCycB1 (S147)/CycB1 (**B**), CycD1/β-actin (**C**) and CycD2/β-actin (**D**) in the cells treated with 2DG (5 mM) with or without metformin (2 mM), after 48 h in culture. * *p* < 0.05 indicates significance when compared to non-treated controls, ♣ *p* < 0.05 indicates significance when compared to control + 2 mM metformin-treated cells and # *p* < 0.05 indicates significance when compared to 2DG (5 mM)-treated cells.

It has been reported that the inhibition of the mTOR pathway reduces cell growth and proliferation [33]. In the present study, the combination treatment with 2DG and metformin inhibited the mTOR pathway in MMECs. We, therefore, studied the effects of treatment with 2DG, metformin and their combination on the modulation of cell-cycle-related proteins (Figure 4).

Treatment of MMECs with 2DG (5 mM) alone significantly increased (~2.1-fold, * *p* < 0.05, Figure 4A,B) the levels of phosphorylated cyclin B1 (pCycB1; S147) when compared to non-treated cells. Metformin (2 mM) and 2DG (5 mM) exposure alone significantly increased cyclicn D2 (CycD2) levels (~1.7-fold and 1.9-fold, * *p* < 0.05, Figure 4A,D), while significantly reducing cyclicn D1 (CycD1) levels (~1.2-fold and 2.4-fold, * *p* < 0.05, Figure 4A,C), when compared to non-treated cells. Treatment of MMECs with a combination of 2DG (5 mM) and metformin (2 mM) for 48 h significantly decreased the levels of pCycB1 (S147; ~1.6-fold and 2.7-fold, ♣ *p* < 0.05 and # *p* < 0.05, Figure 4A,B), CycD1 (~2.7-fold and 3-fold, ♣ *p* < 0.05 and # *p* < 0.05, Figure 4A,C) and CycD2 (~2.6-fold and 3-fold, ♣ *p* < 0.05 and # *p* < 0.05, Figure 4A,D), respectively, when compared to cells treated with either metformin (2 mM) or 2DG (5 mM) alone.

### 2.6. The Effect of the Treatment with a Combination of 2DG and Metformin on the Levels of Anti-Angiogenic TSP1 in MMECs is Independent of 5′ Adenosine Monophosphate-Activated Protein Kinase (AMPK) Activation

**Figure 5:**
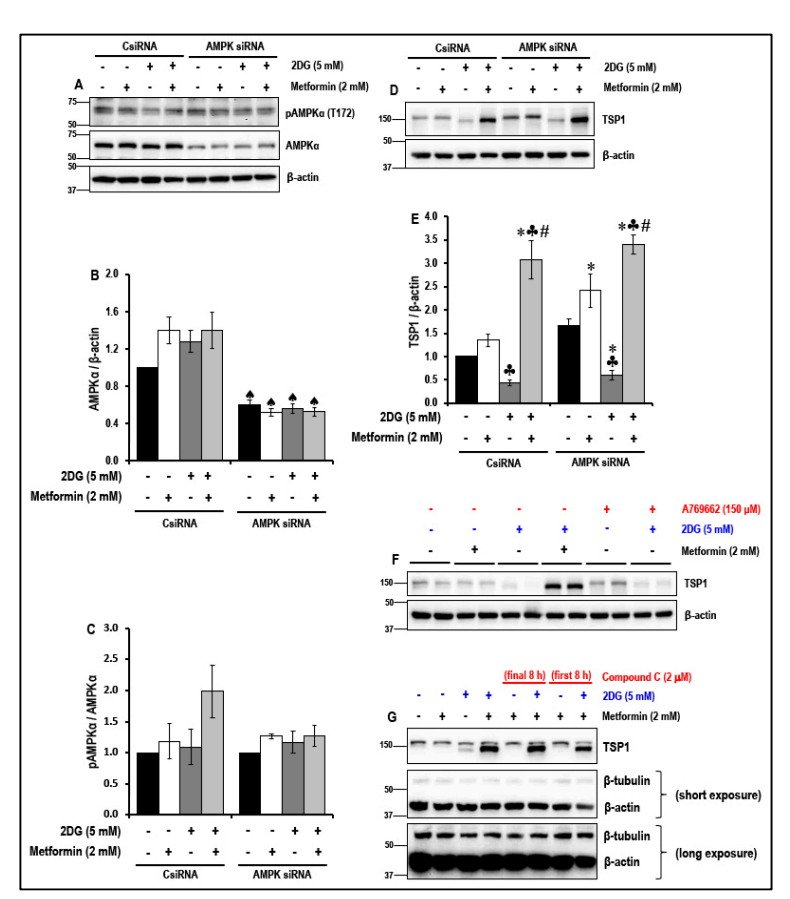
5′ Adenosine Monophosphate-Activated Protein Kinase (AMPK) knockdown and its effect on the levels of TSP1 in metformin-treated normal glucose and 2DG-exposed MMECs (48 h): Representative Western blots (**A**) show the levels of pAMPKα (T172) and AMPKα in control siRNA-treated and AMPK siRNA-treated cells. Bar graphs (**B**) and (**C**) represent the ratio of pAMPKα (T172)/AMPKα and AMPKα/β-actin in the cells (normalized with β-actin loading controls). Representative Western blots (**D**) show the levels of TSP1, respectively, after 48 h in culture. Bar graphs (**E**) represent the levels of TSP1 (normalized with β-actin loading controls) in the cells. Representative Western blots show the levels of TSP1, (**F**) in the presence of metformin and A769662 (150 μM, a putative activator of AMPK), and (**G**) in the presence of metformin and dorsomorphin/compound C (2 μM, a widely used pharmacological inhibitor of AMPK) after 48 h in culture. ♠ *p* < 0.05 indicates significance when the AMPKα levels in AMPK siRNA-treated cells were compared to its respective group control siRNA-treated cells, * *p* < 0.05 indicates significance when compared to non-treated controls, ♣ *p* < 0.05 indicates significance when compared to control + 2 mM metformin-treated cells and # *p* < 0.05 was considered significant when compared to 2DG (5 mM)-treated cells.

Activation of AMPK and downstream AMPK-mediated signaling pathways, reportedly, is key to several of the beneficial effects of metformin. Therefore, the levels of pAMPKα (T172) in the non-treated and metformin-treated MMECs cultured in normal glucose and in the presence of 2DG (5 mM) were studied (Figure 5).

Analysis of Western blot data indicated that there was no significant (*p* > 0.05) increase in the levels of pAMPKα (T172) in the 2DG (5 mM)-exposed metformin (2 mM)-treated MMECs (Figure 5A,C) when compared to the non-treated cells and to cells treated with either metformin (2 mM) or 2DG (5 mM) exposed cells. In order to understand the contribution of AMPK, siRNA against AMPK was used in knockdown experiments and we thus studied whether the level of expression of AMPK was associated with the metformin-treatment-associated upregulation of TSP1 in 2DG-treated MMECs. The data indicate that, knocking down AMPK (Figure 5A,B) markedly increased the levels of TSP1 (Figure 5D,E) in all the groups (irrespective of treatment) when compared to their respective treatment groups exposed to scrambled siRNA (CsiRNA). Even with significant AMPK knockdown (~65% or ~2.7-fold, ♠ *p* < 0.05 when compared to the respective treatment group in CsiRNA treated cells, Figure 5A,B), a combination of 2DG (5 mM) and metformin (2 mM) significantly increased the levels of TSP1 (~5.6-fold, ♣ *p* < 0.05, Figure 5D,E) when compared to cells treated with 2DG alone and with similar levels of AMPK knockdown. We observed that knocking down AMPK had no effect on the metformin (2 mM)-treatment-induced activation of TSP1 in 2DG exposed cells.

Additionally, using a combination of 2DG (5 mM) and A769662 (putative AMPK activator) did not increase the levels of TSP1 (Figure 5F), when compared to cells treated with combination of 2DG (5 mM) and metformin (2 mM). The data also shows that exposure of the cells to dorsomorphin/compound C (2 μM), for the initial 8 h OR final 8 h of the 48 h treatment protocol did not affect the ability of the combination treatment of 2DG (5 mM) and metformin (2 mM) to induce TSP1 (Figure 5G).

### 2.7. Treatment with a Combination of 2DG and Metformin Decreases Cell Proliferation

**Figure 6:**
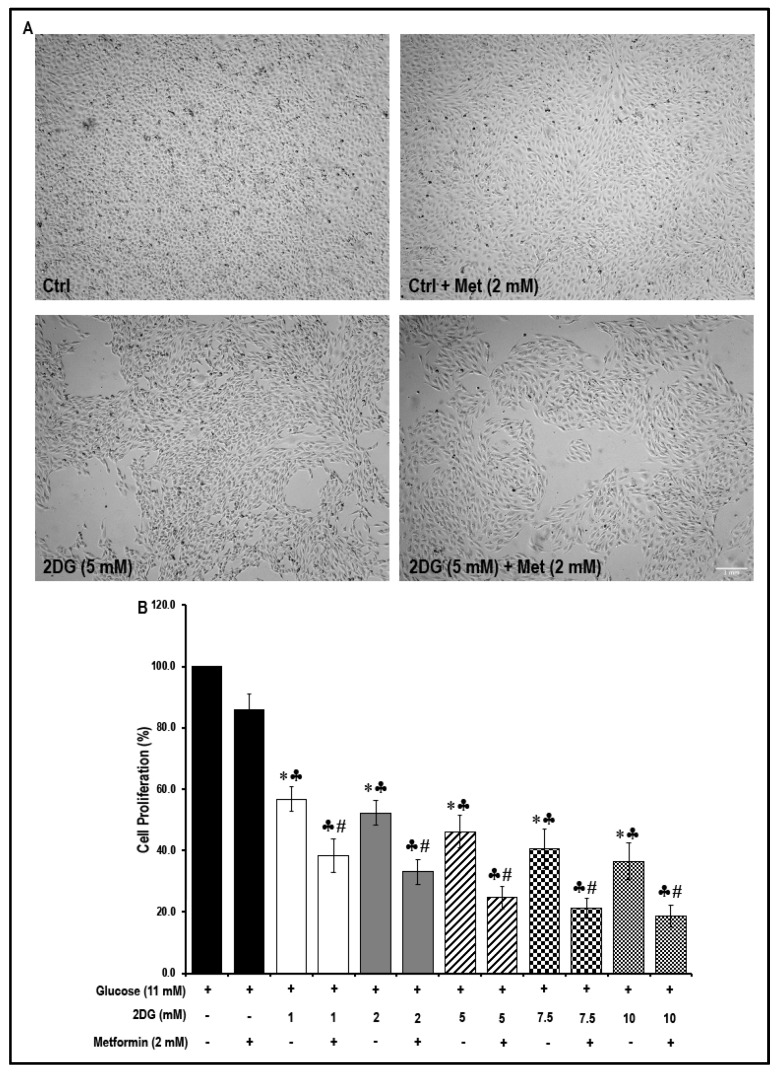
Morphology of MMECs during the different treatment conditions and the effect of metformin treatment on cell proliferation in normal glucose- and 2DG-exposed MMECs (48 h): Representative microscopic images (**A**; from four experiments) show the morphology of MMECs under the different treatment conditions after 48 h in culture. The bar graph (**B**) shows the rate of cell proliferation in MMECs treated with varying concentrations of 2DG with or without metformin (2 mM), after 48 h in culture. Non-treated cells and cells treated with metformin (2 mM) alone were used as suitable controls. * *p* < 0.05 was considered significant when compared to non-treated controls, ♣ *p <* 0.05 was considered significant when 2DG treatment was compared to the metformin (only)-treated cells. # *p <* 0.05 was considered significant when the combination treatment, 2DG + Met, was compared to is respective 2DG (only)-treated cells. For example, # *p* < 0.05 was considered significant when; 2DG (1 mM) + Met (2 mM) was compared to 2DG (1 mM); 2DG (2 mM) + Met (2 mM) was compared to 2DG (2 mM); 2DG (5 mM) + Met (2 mM) was compared to 2DG (5 mM). Scale bar = 1000 μm.

The Western blot data revealed that treatment with a combination of 2DG and metformin in MMECs inhibited the Akt/mTOR pathway and cell-cycle-related proteins and these data suggested that metformin inhibited cell proliferation. To test this hypothesis, an MTS assay was performed. Cell morphology (Figure 6A) was not altered under the different treatment conditions and neither was there a significant change in the rate of cell proliferation in metformin (2 mM)-treated MMECs when compared to non-treated controls.

However, exposure to all of the different concentrations of 2DG (1 mM, 2 mM, 5 mM, 7.5 mM and 10 mM) significantly reduced cell proliferation (Figure 6B) when compared to non-treated controls. Furthermore, treatment with 2 mM metformin in the 2DG exposed cells resulted in a further decrease (Figure 6B) in cell proliferation. While exposure to 2DG (5 mM) alone caused a significant decrease in cell proliferation (~54%, * *p* < 0.05, Figure 6B), a combination of 2DG (5 mM) and metformin (2 mM) caused a greater decrease in cell proliferation by ~75% (* *p* < 0.05, Figure 6B) when compared to non-treated controls. There was a significant decrease in cell proliferation (~1.4-fold, # *p* < 0.05, Figure 6B) in 2DG (5 mM) + metformin (2 mM)-treated cells when compared to cells treated with 2DG (5 mM) alone.

**Figure 7:**
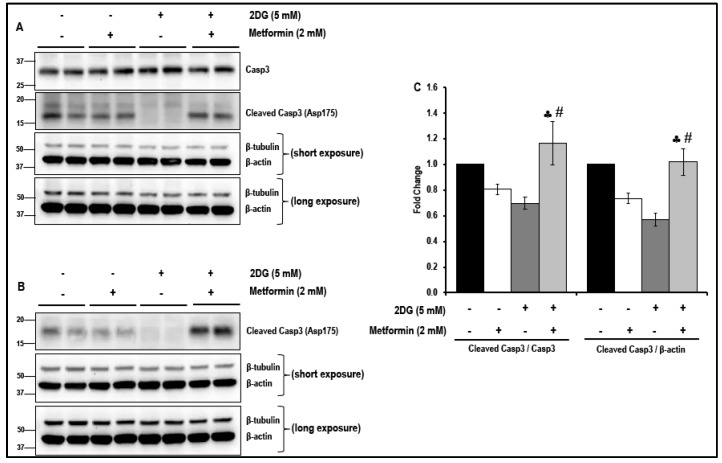
The levels of caspase-3 (Casp3) and cleaved caspase-3 in metformin-treated normal glucose- and 2DG-exposed MMECs (48 h): Representative Western blots, (**A**) shows the levels of total caspase-3 and cleaved caspase-3 (detected using the anti-caspase-3 antibody; Cat # 9665; Cell Signaling Technology, Danvers, MA, USA) and (**B**) shows the levels of cleaved caspase-3 (using the anti-cleaved caspase-3 antibody; Cat # 9664; Cell Signaling Technology, Danvers, MA, USA), in cells treated with 2DG (5 mM) in the presence or absence of metformin (2 mM), after 48 h in culture. Non-treated cells and cells treated with metformin (2 mM) alone were used as suitable controls. Bar graphs (**C**) represent the ratio of (normalized against β-actin loading controls) cleaved caspase-3/caspase 3 and cleaved caspase-3/β-actin in the cells treated with 2DG (5 mM) with or without metformin (2 mM), after 48 h in culture. ♣ *p* < 0.05 indicates significance when compared to control + 2 mM metformin-treated cells and # *p* < 0.05 indicates significance when compared to 2DG (5 mM)-treated cells.

Western blot analysis indicates no significant differences either in the levels of cleaved caspase-3 in cells treated with either 2DG or metformin alone or in the levels of cleaved caspase-3 in cells that were exposed to a combination of 2DG and metformin when compared to the non-treated cells. These data, therefore, eliminate the likelihood of toxic effects of the drugs at the concentration that were used. (Figure 7A–C). However, in contrast, the increase in the levels of cleaved caspase-3 was significant in cells treated with a combination of 2DG and metformin when compared to cells treated with either 2DG or metformin alone (Figure 7A–C). It must be noted that two different antibodies were used (see Section 4.1 on ‘Antibodies’ for details). The primary anti-cleaved caspase-3 detected only the cleaved form of caspase-3 (hence represented as a ratio of cleaved caspase-3/β-actin), while the primary anti caspase-3 detected the total, as well as cleaved forms of caspase-3, and hence, the ratio of cleaved caspase-3/caspase-3 was presented).

### 2.8. Treatment with a Combination of 2DG and Metformin Inhibits Cell Migration and Tubulogenesis in MMECs

**Figure 8:**
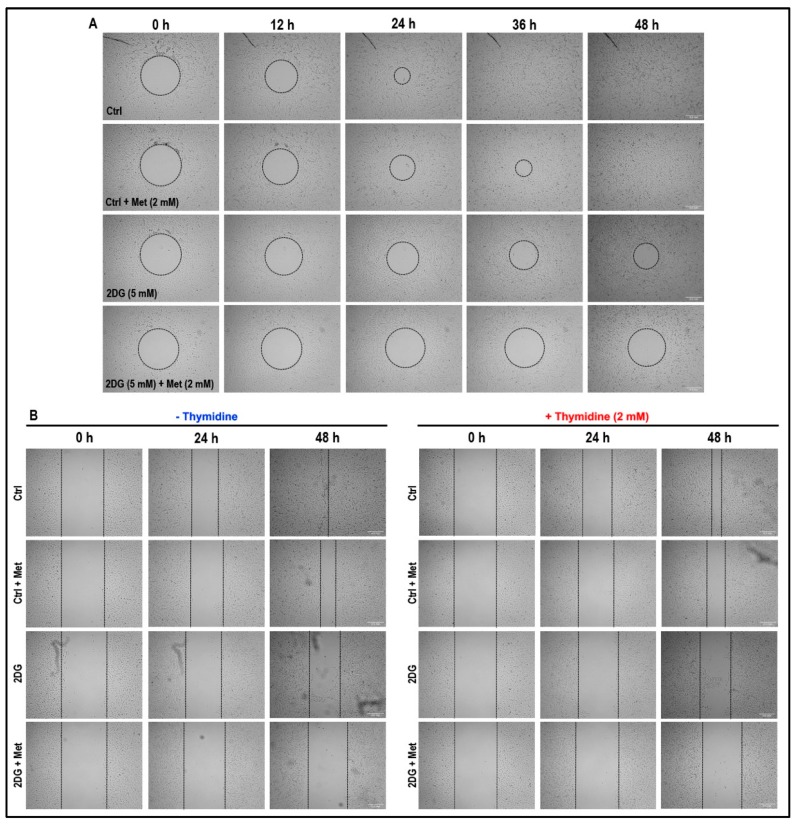
Effect of metformin treatment on cell migration in normal glucose- and 2DG-exposed MMECs: Representative microscopic images (**A**; from four experiments) from a ‘Radius’ cell migration assay show the rate of cell migration under the different treatment conditions at zero time (0 h) and after 12 h, 24 h, 36 h and 48 h in culture. Representative microscopic images (**B**; from three experiments) from Cytoselect cell migration assay show the rate of cell migration under the different treatment conditions in the absence or presence of thymidine (2 mM) at zero time (0 h) and after 24 h and 48 h in culture. Scale bar = 500 μm.

We then investigated whether the combination treatment with 2DG (5 mM) plus metformin (2 mM) could influence the cell migration. In a Radius™ cell migration assay (Figure 8A), non-treated cells achieved complete gap/wound closure at 36 h after treatment.

Although, treatment with metformin (2 mM) alone decreased cell migration, complete gap/wound closure was observed after 48 h (Figure 8A). Similarly, although treatment with 2DG (5 mM) alone further decreased the rate of cell migration (when compared to non-treated cells and cells treated with metformin alone), a marked reduction in the wound/gap area was observed after 48 h of treatment (Figure 8A). However, the treatment with a combination of 2DG (5 mM) and metformin (2 mM) had a dramatic effect and significantly reduced the rate of cell migration with no significant gap/wound closure even after 48 h of treatment when compared to non-treated cells, and cells treated with either 2DG (5 mM) or metformin (2 mM) alone (Figure 8A). The wound closure observed in Figure 8A is an outcome of cell migration and proliferation.

In order to assess the effect of our treatment on cell migration alone, the cell migration assay was performed in the absence or presence of thymidine (2 mM). Thymidine (2 mM) exposure delayed the wound healing process under all treatment conditions (Figure 8B). The wound closure observed in Figure 8B is an outcome of cell migration since cell proliferation is blocked by thymidine exposure.

**Figure 9:**
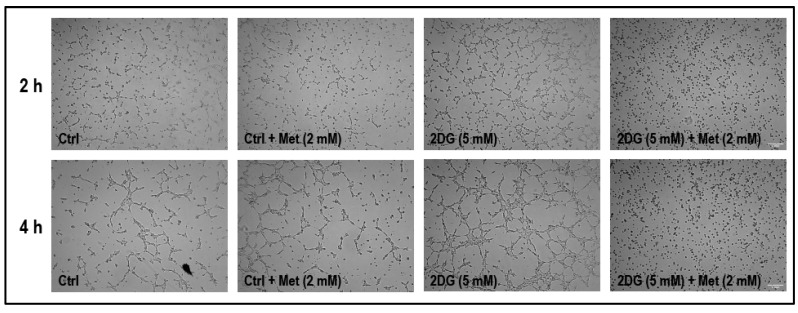
Representative microscopic images (from three experiments) from matrigel tubulogenesis assay shows the tubulogenic capacity of the cells under the different treatment conditions. It must be noted that the MMECs were treated for 24 h on 60 mm dishes prior to tubulogenesis assay. After 24 h in culture, the MMECs were trypsinized and harvested and seeded (20,000 cells/well) onto the matrigel in their respective treatment media/conditions. We intended to image the cells after 2 h, 4 h, 6 h, 12 h and 24 h (to match the 48 h time point for the other experiments) on a matrigel. However, during initial experiments, it was noted that the cells did not grow well beyond 4–5 h on a geltrex matrix, hence images of the cells were captured using a camera mounted on an inverted light microscope after 2 h and 4 h on a geltrex matrix. Scale bar = 1000 μm.

We then investigated whether treatment with the combination of 2DG (5 mM) and metformin (2 mM) would influence tubulogenic capacity of cells on a basement membrane matrix (see Section 4.7 on ‘tubulogenesis assay’ for details of the experimental protocol). The cells were initially treated and cultured in 60 mm petri dishes for 36 h before being transferred onto the basement membrane matrix. The tube/network formation dissipated within 6 h after being transferred onto the basement membrane matrix in non-treated cells and hence the experiment was restricted to 4 h. Matrigel-based tubulogenesis assay revealed a marked increase in tubulogenic capacity in cells treated with 2DG (5 mM) alone after 2 h and 4 h in culture (Figure 9). After 2 h and 4 h in culture, the tubulogenic capacity was completely abolished in cells treated with a combination of 2DG (5 mM) and metformin (2 mM) when compared to non-treated cells, and cells treated with either 2DG (5 mM) or metformin (2 mM) alone (Figure 9).

### 2.9. Treatment with a Combination of 2DG and Metformin Differentially Modulates the Levels of OCTs

**Figure 10:**
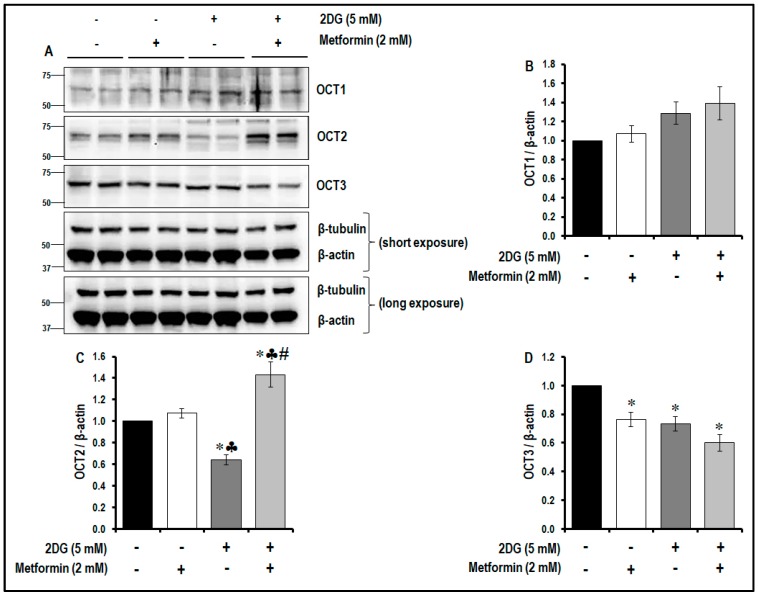
The levels of organic cation transporters-1, 2 and 3 (OCT1, OCT2 and OCT3) in metformin-treated normal glucose- and 2DG-exposed MMECs (48 h): Representative Western blots (**A**) show the levels of OCT1, OCT2 and OCT3 metformin (2 mM)-treated normal glucose- and 2DG (5 mM)-exposed MMECs. Non-treated cells and cells treated with metformin (2 mM) alone were used as suitable controls. Bar graphs represent the ratio of (normalized against β-actin loading controls) OCT1/β-actin (**B**), OCT2/β-actin (C) and OCT3/β-actin (**D**) in the cells treated with 2DG (5 mM) with or without metformin (2 mM), after 48 h in culture. * *p* < 0.05 indicates significance when compared to non-treated controls, ♣ *p* < 0.05 indicates significance when compared to control + 2 mM metformin-treated cells and # *p* < 0.05 indicates significance when compared to 2DG (5 mM)-treated cells.

It is evident that the combination treatment of 2DG and metformin significantly increased TSP1 levels while decreasing the levels of pVEGFR2 (Y1175), inhibited the mTOR pathway and decreased cell proliferation, migration and tubulogenesis in MMECs. In order to determine the most likely mode of entry of metformin into the cells, we examined the levels of OCT1 and OCT2 and OCT3 in the cells. OCT1, OCT2 and OCT3 were detected by Western blotting the cells. The expression of OCT1 did not change in cells treated with metformin alone, 2DG alone or in the combination treatment (Figure 10A,B) when compared to non-treated controls; however, the expression levels of OCT3 significantly decreased in cells treated with metformin alone, 2DG alone and also in the combination treatment protocol (Figure 10A,D) when compared to non-treated controls. Interestingly, the changes in OCT2 were more pronounced in the treated cells. Treatment with metformin alone did not affect the expression of OCT2, but 2DG treatment alone significantly decreased OCT2 levels (~1.6-fold, # *p* < 0.05, Figure 10A,C) the cells when compared to non-treated cells. On the other hand, the combination treatment using 2DG (5 mM) and metformin (2 mM) significantly increased OCT2 levels (Figure 10A,C) when compared to non-treated controls (~1.4-fold), cells treated with metformin alone (~1.3-fold) and cells treated with 2DG alone (~2.2-fold).

## 3. Discussion

**Figure 11:**
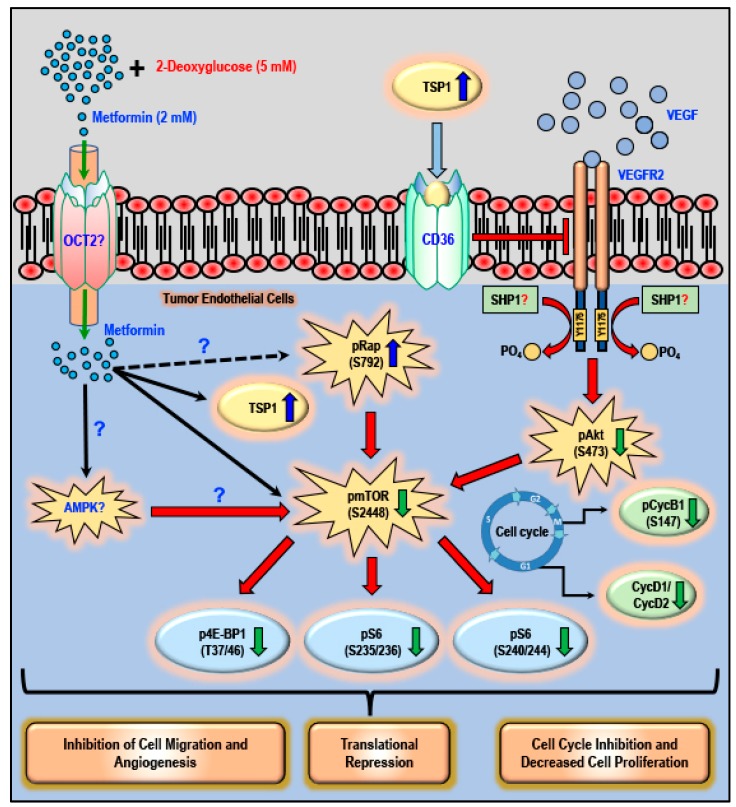
Targeting angiogenesis in tumor endothelial cells with a combination of metformin and 2DG: The schematic illustrates the possible cellular sites of action when using a combination of metformin and 2DG in tumor endothelial cells. It is possible that metformin accumulates in the tumor endothelial cells via OCT2 and requires futher thorough investigation. A combination of 2DG (5 mM) and metformin (2 mM) causes a robust increase in the levels of anti-angiogenic TSP1, which then binds to its receptor CD36 and negatively regulates the phosphorylation of VEGFR2 at Y1175. A decrease in the levels of pVEGFR2 (Y1175), in turn, reduces the levels of phospho-Akt (S473). The combination treatment (DG and metformin) also increases the levels of phospho-Rap (S792). The increase in the levels of inhibitory pRap (S792) and decrease in pAkt (S473) subsequently decreases the levels of phospho-mTOR (S2448), resulting in the decrease in the levels of downstream phospho-4E-BP1 (T37/46) and phospho-S6 ribosomal protein (S235/236 and S240/244). The combination treatment also reduces the levels of cell-cycle-associated phospho-CycB1 (S147), CycD1 and CycD2. These molecular events associated with the combination therapy (2DG + metformin) result in translational repression, inhibition of cell migration and angiogenesis and the inhibition of cell cycle and proliferation. Further investigations are warranted to ascertain or rule out the possible role of AMPK in the effect that we have seen on the levels of phospho-Rap and inhibition of the mTOR pathway when using a combination of 2DG and metformin.

The data presented in this study documents that when compared to using either 2DG or metformin alone as a potential anti-cancer treatment regimen, using the combination of the glycolytic inhibitor 2DG (5 mM) and metformin (2 mM) activates anti-angiogenic TSP1, inhibits the Akt/mTOR pathway and decreases the levels of cyclins, leading to inhibition of tubulogenesis, cell proliferation and migration in cancer endothelial cells (Figure 11).

In a hypoxic tumor microenvironment, angiogenic TECs are characterized by high rates of glycolysis and glutaminolysis, which provide the required biosynthetic intermediates and the energy to support important cellular processes and functions without coupling to oxidative phosphorylation [34]. TECs maintain this metabolic preference even under normoxic/aerobic conditions [34]. The cellular-respiration-independent metabolism in angiogenic TECs supports cell survival, proliferation and migration, whereby the newly formed blood vessel contributes to tumor survival, growth and metastasis [34,35,36,37]. Additionally, upon VEGF stimulation, TECs double their glycolytic flux to meet the increased demand for energy and biosynthetic molecules for cellular processes [38,39]. Although clinically approved and tested, pharmacological inhibition of VEGF has limited benefits as an anti-angiogenic therapeutic strategy in cancers since tumors are known to acquire resistance to a drug regimen that solely targets the VEGF pathway [23]. Hence, a better and refined approach would be to target TEC metabolism and block glycolysis that should have anti-endothelial and anti-angiogenic effects, and thus be more efficient in the treatment of different forms of cancers [40]. Agents such as 3-(3-pyridinyl)-1-(4-pyridinyl)-2-propen-1-one (glycolytic inhibitor of fructose-2,6-bisphosphatase 3/phosphofructokinase-2) [41,42], alpha-cyano-4 hydroxycinnamic acid (inhibitor of mono-carboxylate transporter) [43,44] and dichloroacetate (inhibits pyruvate dehydrogenase kinase) [45] exert their anti-cancer potential in different cancers by targeting the aberrant metabolism in cancer cells. The glycolytic inhibitor, 2DG has been shown to effectively reduce tumor growth and survival in different kinds of tumors [46]. 2DG simultaneously targeted cancer and endothelial cells to suppress the growth of neuroblastomas in mice [47]. However, using glycolytic inhibitors alone may cause a metabolic switch towards glutaminolysis, thus supporting cancer cell survival and growth [48].

Low concentrations (50 μM) of metformin are vasculoprotective, improve endothelium-dependent vasodilation in blood vessels from diabetic mice and protect endothelial cells against senescence via a mechanism(s) that is dependent on the expression of the anti-aging gene product, the de-acetylase sirtuin 1, while higher concentrations may have detrimental effects on cell metabolism and survival [14,49,50,51]. Metformin is not metabolized and is excreted unchanged and thus selective accumulation in some types of cancer may explain the reported variable therapeutic efficacy of metformin to have a direct action to reduce tumorigenesis in some patients. Interestingly, lower concentrations of metformin are required to induce cell arrest in in vivo studies with normoglycemic versus hyperglycemic mice with ovarian cancer as well as in in vitro studies with human ovarian cancer cell lines [52]. The efficacy of metformin in breast cancer cells is markedly reduced with high glucose concentrations and is associated with increased aggressiveness of the cancer cells, depending on the glucose concentration [15]. Treatment with metformin reportedly caused suppression of tumor angiogenesis by targeting the human epidermal growth factor receptor-2 /hypoxia inducible factor-1 alpha/VEGF (HER2/HIF-1α/VEGF) secretion axis in different breast cancer cell lines [53]. In women with polycystic ovarian syndrome, treatment with metformin inhibited angiogenesis as a result of the increase in the serum levels of anti-angiogenic TSP1 [28].

In order to avoid the potential limitations of using either 2DG or metformin alone in the treatment of cancer, in vitro studies have demonstrated that a combination of 2DG and metformin has a synergistic effect and was found to be more effective in the treatment of various types of cancers [20,54,55,56,57]. In our current study, we used this strategy and studied the effect of a combination of metformin and 2DG on angiogenic and anti-angiogenic markers, the mTOR pathway, cell proliferation, migration and tubulogenic capacity of TECs (the MMECs).

Based on preliminary experiments performed in our laboratory, we observed that concentrations of metformin from 25 μM to 1 mM did not activate TSP1 in glucose-starved cells. The data we now present indicates that treatment with 2 mM metformin resulted in a robust increase in the levels of TSP1 in glucose-starved MMECs after 24 and 48 h in culture (Figure 1). As it is not possible to clinically subject an individual to glucose starvation and in order to allow the results of this study to translate to a clinical scenario, we chose to use the glycolytic inhibitor, 2DG, and experimental protocols were designed to examine whether a combination of 2DG and metformin (2 mM) would bring about a similar effect in raising the levels of TSP1 as that observed with metformin (2 mM) treatment in glucose-starved MMECs. Western blot analysis indeed revealed a significant increase in the levels of TSP1, but only in cells treated with a combination of 2DG and metformin (2 mM) and not in cells treated with 2DG or metformin alone (Figure 2). The anti-angiogenic TSP1 is known to bind to its receptor, CD36, and inhibit growth-factor-induced angiogenic signaling [32,58,59,60]. TSP1 exerts its anti-angiogenic effect through TSP1-CD36 binding and association which leads to the recruitment of SHP1 to the VEGFR2 receptor with subsequent VEGFR2 de-phosphorylation and subsequent inactivation of VEGF/VEGFR2 signaling [32]. In our present study, we have shown an increase in TSP1-CD36 co-localization associated with a significant decrease in the levels of pVEGFR2 (Y1175), which we observed only in MMECs treated with a combination treatment of 2DG and metformin (Figure 2). The MMECs that we used overexpress VEGF; the high levels of VEGF should be adequate to trigger VEGFR2 phosphorylation in the cells. The increase in VEGFR2 phosphorylation in the presence of 2DG alone (Figure 2) correlates to the increase in tubulogenesis in the cells treated with 2DG (Figure 9). However, the decrease in pVEGFR2 (Y1175) levels that we observed with the combination treatment of metformin and 2DG is apparently due to the de-phosphorylation of VEGFR2 as a result of TSP1-CD36 association and possible Src-homology 2-containing tyrosine phosphatase-1 (SHP1) recruitment, which correlates to the decrease in tubulogenic capacity of the cells treated with a combination of 2DG (5 mM) and metformin (Figure 9) [32].

VEGF/VEGFR2 signaling is known to trigger the Akt/mTOR pathway and angiogenesis in endothelial cells [61,62]. Deregulation of the PI3K/Akt/mTOR pathway contributes to activation of cell proliferation, adhesion, migration, invasion, metabolism, and survival in many cancers [61,63]. The mTOR inhibitor rapamycin inhibits VEGF-driven angiogenesis and decreased phosphorylation of both Akt and S6-ribosomal protein [62]. In the present study, the combination treatment with 2DG and metformin, associated increase in TSP1 and decrease in the levels of pVEGFR2 (Y1175) resulted in a significant decrease in the levels of pAkt (S473) and subsequent decrease in the levels of pmTOR (S2448) (Figure 3). In addition, the combination of 2DG and metformin increased the levels of pRap (S792), an endogenous negative regulator of mTOR activation [64] (Figure 3). This is in accordance with our previous study where we observed a significant increase in the levels of pRap (S792) levels and decrease in the levels of pAkt (S473) and and mTOR (S2448) in the metformin-treated glucose-starved MMECs [16]. Treatment with a combination of 2DG and metformin in MMECs also decreased the levels of p4E-BP1 (T37/46), pS6 (S235/236) and pS6 (S240/244), downstream of mTOR inhibition (Figure 3). A decrease in the phosphorylation of 4E-BP1 and S6-ribosomal protein suppresses overall protein synthesis and inhibits the translation of mRNA transcripts that encode proteins involved in cell cycle progression, ribosomal proteins and elongation factors required for translation [65,66]. Metformin [54], the PI3K/Akt inhibitor-LY294002-and the mTOR inhibitor-rapamycin-have previously been shown to inhibit cyclin D1 protein expression [67]. Furthermore, the treatment with a combination of 2DG and metformin in MMECs resulted in a significant decrease in the levels of cell-cycle-related proteins pCycB1 (S147), CycD1 and CycD2 (Figure 4). The increase in the levels of TSP1, its co-localization with CD36, followed by decrease in the levels of pVEGFR2 (Y1175), inhibition of the Akt/mTOR pathway and decrease in the levels of cell-cycle-related proteins, can be correlated to the decrease in cell proliferation (Figure 6), migration (Figure 8) and tubulogenic capacity (Figure 9) that we observed in the MMECs treated with a combination of 2DG and metformin (Figure 11). Although not conclusive, our data indicates that it is most likely that the effects of metformin are intracellular as, “inward” OCT transporters are expressed in MMECs and an upregulation of the OCT2 transporter was detected in the the cells treated with a combination of 2DG and metformin (Figure 10A,C). However, other OCTs and also transporters (PMAT and MATE) that support the extrusion of metformin from within the cell may also play important roles and require additional thorough inverstigations. The elucidation of the molecular mechanism of TSP1 upregulation in the MMECs with a combination of 2DG (5 mM) and metformin (2 mM) also requires more extensive studies and notably, the role of pathways that play a role in TSP1 activation such as TGF-β1, p38 MAPK, NF-κB and Erk1/2/Erk5 [28,68].

Activation of AMPK in endothelial cells is essential for angiogenesis [69]. Additionally, VEGF-mediated activation of AMPK has been shown to promote angiogenesis [70]. It is widely accepted that the majority of the cellular effects of metformin are mediated by the activation of AMPK [5,10,71]. However, the anti-cancer effects of mM levels of metformin may depend, at least in part, on non-AMPK-dependent mechanisms [16,72]. Metformin-mediated AMPK activation, as well as inhibition of angiogenesis, has been reported [73,74,75,76]. We have previously shown that the reversal of autophagy following treatment with 2 mM metformin was independent of AMPK levels [16]. In the present study, we, therefore, studied whether knocking down AMPK would negate the effects of metformin on TSP1 levels in MMECs exposed to 2DG. Treatment of the cells with 2DG or metformin alone or a combination of 2DG and metformin did not cause a significant change in AMPK activation possibly because the cells can use alternative mechanisms to maintain ATP levels within the cell [34,36,38]. In the current study, AMPK knockdown (~65%) did not reverse the effect of the combination of 2DG and metformin on the expression levels of TSP1 (Figure 5). Activation of AMPK in endothelial cells is essential for angiogenesis and in our study, AMPK knockdown alone resulted in an increase in TSP1 levels in the non-treated control cells, as well as in cells treated with metformin alone. Furthermore, we used a putative AMPK activator, A769662 [77], in combination with 2DG in order to investigate whether a known AMPK activator could bring about effects in these cells similar to what we had observed when using 2DG and metformin in combination. When the combination of 2DG and metformin increased the TSP1 levels in the MMECs, a combination of 2DG and A769662 failed to cause a similar effect on the expression of TSP1 (Figure 5). Additionally, the MMECs treated with metformin alone (2 mM) or a combination of 2DG (5 mM) and metformin (2 mM) were also exposed to dorsomorphin/compound C (2 μM), a pharmacological inhibitor of AMPK, for the initial 8 h or final 8 h of the 48 h treatment protocol. The data shows that the exposure to compound C did not affect the ability of the combination treatment of 2DG (5 mM) and metformin (2 mM) to induce TSP1 (Figure 5G). It must be noted that compound C potently inhibits several other kinases, other than AMPK, and, therefore, the data derived using compound C cannot be deemed conclusive in order to rule out the role of AMPK on TSP1 upregulation in this study [78,79]. This data is, however, indicative that the activation of TSP1 observed in cells treated with a combination of 2DG and metformin are, based on the compound C data, independent of AMPK activation. A potential limitation of our study is that we have not clearly defined an alternate mechanism to explain the upregulation of TSP1 in the cells treated with a combination of 2DG (5 mM) and metformin (2 mM), nonetheless, we demonstrate that the metformin-related effects that we observed are independent of AMPK.

A limitation of our study is that in the cell culture protocol, high concentrations of metformin (2 mM) and 2DG (5 mM) were required to achieve the effects that we have observed. Since endothelial cells are glycolytic by nature, in a clinical scenario, a genuine concern would be that a systemic administration of a high concentration of 2DG would compromise the viability of the entire endothelial cell lining that is in immediate contact with blood. Using HUVECs and lung-derived human microvascular endothelial cells (HMVEC-L), it has been shown that 2DG treatment in these cells significantly reduced growth and induced cytotoxicity (starting at 600 μM) in a dose-dependent manner (60 μM, 600 μM, 6 mM and 9 mM) [80]. Furthermore, intravenous administration of 2DG caused, although transient and not severe, effects similar to hypoglycemia, possibly because glucose is the primary fuel for brain and 2DG is a competitive inhibitor of glucose, inhibiting glycolysis [81]. However, it must be noted that both HUVECs and HMVEC-L cells were significantly more sensitive to the cytotoxic effects of a low dose (0.6 mM) of 2-DG than a panel of human cancer cells (HT29, CAKI-1, MDA-MB-231 and HT-1080) [80]. The tumor endothelial cells are addicted to glycolysis and are metabolically similar to cancer cells. When used as an oral medication for the treatment of type 2 diabetes, plasma levels of metformin are in the low μM range [8]. It remains unclear whether metformin can accumulate in cancer cells to reach such high concentrations, but, as previously discussed, the differential expression of the cation transporters that regulate the absorption and extrusion could result in the accumulation of metformin in cancer cells and thus explain the therapeutic efficacy of metformin in certain types of cancer [13]. The accumulation of metformin to high micromolar/millimolar levels would also result in the inhibition of mitochondrial complex 1, resulting in decreased mitochondrial respiration [82,83]. Its is also possible that although using the drugs at high concentrations may be effectively anti-angiogenic in cell-based in vitro studies, the same may not be true in vivo since a scenario of vessel disintegration in the tumors, as opposed to tumor vessel normalization, may facilitate cancer cell escape and metastasis [84,85]. Further studies are, therefore, required to optimize the dosage of 2DG and of metformin (using doses appropriate for the treatment of type 2 diabetes) and to determine whether the concentrations found to be effective in pre-clinical studies can be safely applied in the clinical scenario. Alternatively, if such high concentrations of 2DG or metformin are necessary for the treatment of cancer then pre-clinical and clinical studies are warranted to shed light on 1) whether properties specific to cancer/neoplastic cells can be exploited and 2) drug delivery technology (such as nanoparticle delivery systems) can be used to achieve the targeted or localized delivery and accumulation of drugs specifically into the cancer cells with maximum efficacy as a therapeutic strategy.

Our previous observations [16] and the findings from the current study reveal the potential of using a combination of metformin and glycolytic inhibitors to target TECs as an effective anti-endothelial and anti-angiogenic strategy to curb tumor growth and combat various cancers. Combinatory drug approaches, when compared to monotherapy, tend to be more effective in the treatment of different cancers since drug combinations (1) reduce the effective dosage required of the individual drugs to bring about a positive outcome, (2) reduce the potential for the development of resistance to an individual drug, (3) increase the sensitivity of cancers to chemo- and radiotherapy approaches and (4) improve overall prognosis.

## 4. Materials and Methods

### 4.1. Antibodies, Biochemicals, Chemicals and Reagents

Metformin (Cat # D150959), 2-deoxyglucose (2DG; Cat # D8375), dorsomorphin/compound C (Cat # P5499), thymidine (Cat # T9250) and all the biochemicals, chemicals and reagents used in the current study were of analytical grade, and unless otherwise stated, were purchased from Sigma-Aldrich, Inc. (St.Louis, MO, USA).

Primary anti-thrombospondin-1 antibodies were purchased and tested from two sources (1) anti-TSP1 (Cat # 37879) from Cell Signaling Technology, Inc. (Danvers, MA, USA) and (2) anti-TSP1; Cat # ab85762) from Abcam Inc. (Cambridge, MA, USA). Primary, anti-pAMPK (T172; Cat # 2531), anti-AMPK (Cat # 2532), anti-pAkt (S473; Cat # 4060), anti-Akt (Cat # 4691), anti-pmTOR (S2448; Cat # 5536), anti-mTOR (Cat # 2983), anti-p4E-BP1 (T37/46; Cat # 2855), anti-4E-BP1 (Cat # 9644), anti-pS6 (S235/236; Cat # 4858), anti-pS6 (S240/244; Cat # 5364), anti-S6 ribosomal protein (Cat # 2317), anti-pVEGFR2 (Y1175; Cat # 2478), anti-VEGFR2 (Cat # 2479), anti-pCyclin B1 (S147; Cat # 4131), anti-Cyclin B1 Cat # 4135), anti-Cyclin D1 (Cat # 2978), anti-Cyclin D2 (Cat # 3741), anti-cleaved caspase-3 (Cat # 9664), anti-caspase-3 (Cat # 9665), anti-β-actin (Cat # 3700) and anti-β-tubulin (Cat # 2128) antibodies and secondary, HRP-linked antibodies for Western blotting (anti-rabbit IgG, Cat # 7074 and anti-mouse IgG, Cat # 7076) and Alexa-Fluor tagged antibodies for immunofluorescence studies (anti-rabbit Alexa-Fluor 555, Cat # 4413 and anti-mouse Alexa-Fluor 488, Cat # 4408) were purchased from Cell Signaling Technology, Inc. (Danvers, MA, USA). Primary anti-OCT1 (anti-SLC22A1; Cat # ab55916), anti-OCT2 (anti-SLC22A2; Cat # ab170871) and anti-OCT3 (anti-SLC22A3; Cat # ab183071) were purchased from Abcam Inc. (Cambridge, MA, USA). The anti-CD36 primary antibody (Cat # sc-7309) was sourced from Santa Cruz Biotechnology, Inc. (Dallas, TX, USA).

### 4.2. Endothelial Cell Culture

Mouse microvascular endothelial cells (MMECs, CRL-2460, MS1-VEGF) were sourced from American Type Culture Collection (ATCC, Manassas, VA, USA) and serially passaged (p5–p10) for the study in Dulbecco’s Modified Eagle’s Medium (DMEM; Invitrogen, Carlsbad, CA, USA), at 11 mM glucose concentration, supplemented with 5% FBS (Sigma-Aldrich, St.Louis, MO, USA), in a humidified atmosphere with 5% CO_2_ at 37 °C, as previously described [16,49]. A value of 11 mM glucose (normal glucose levels for MMECs) is based on random plasma glucose measurements established from C56/BL mice [86]. Primate VEGF-121 was overexpressed in the MS1 pancreatic microvasculature endothelial cell line (CRL-2279; ATCC, Manassas, VA, USA) to derive the MS1-VEGF/MMEC line [29]. MMECs reportedly generate well-differentiated angiosarcomas in nude mice [29]. Additionally, these cells have been used to investigate signal transduction pathways involved in tumorigenesis and angiogenesis and are, therefore, considered appropriate for the proposed studies on the putative anti-angiogenic effects of metformin [29,87,88].

### 4.3. Cell Treatments

Recent studies from our laboratory [16], showed that only in the glucose-starved MMECs, 2 mM, but not 50 μM, metformin, effectively reversed the glucose-starvation-induced pro-survival mechanisms in cancer endothelial cells (the MMECs) via the inhibition of autophagy and the Akt/mTOR pathway, resulting in G2/M cell cycle arrest and increased cell death [16]. Concentration-dependent studies, as described previously [16], on the effect of increasing concentrations of metformin from 25 μM to 20 mM (25, 50, 100, 250, 500 μM and 1, 2, 5, 10, 20 mM) revealed that metformin did not activate TSP1 in normal glucose-exposed MMECs. However, in glucose-starved MMECs, while lower concentrations of metformin (25 μM to 1 mM) did not activate TSP1, treatment with 2 mM metformin caused a robust increase in the levels of TSP1 in glucose-starved MMECs after 48 h in culture. Hence, 2 mM metformin was chosen as the working concentration for this study. Furthermore, mM concentrations of the drug have also been used for many of the in vitro investigations of the putative pathways that might mediate the anti-cancer effects of metformin. Although we understand that the concentration of metformin (2 mM) that we have used in this study is much higher when compared to the reported peak plasma concentrations of metformin after oral administration of the highest dose of metformin [8,89], when it is used for the treatment of type 2 diabetes, it has been reported that in cancer cells such as the BT-20 breast cancer cells, metformin uptake is enhanced by more than 13-fold and was found to be associated with an overexpression of the OCT3 cation transporter [13]. In addition, the anti-proliferative potency of metformin was also found to be 4-fold higher in OCT3 overexpressing BT-20 cells when compared to the cation-selective transporter-deficient BT-20 cells [13]. These findings raise the possibility that the cellular accumulation of metformin may occur in some tumors such that the cancer cell is exposed to comparatively higher levels of the drug and thus should explain the apparent selective toxicity of metformin. For the initial part of the study, MMECs (p5–p10) were glucose-starved for 24 h and 48 h in the absence or presence of metformin (50 μM or 2 mM), as described previously [16]. Normal glucose (11 mM) [86]-exposed cells in the absence or presence of metformin were considered as normal controls.

In addition to the normal glucose levels (11 mM) in the treatment media, MMECs (p5–p10) were exposed to varying concentrations of 2DG (1 mM, 2 mM, 5 mM, 7.5 mM and 10 mM) in the absence or presence of metformin (2 mM) for 48 h. In normal glucose-exposed cells, without 2DG, in the absence or presence of metformin were considered as suitable normal controls. MMECs were also subjected to 2DG treatment for 48 h in the absence or presence of the AMPK activator, A769662 (Cat # sc203790; 150 μM) sourced from Santa Cruz Biotechnology, Inc. (Dallas, TX, USA). Normal glucose (11 mM)-exposed cells in the absence or presence of A769662 were considered as comparable controls.

Additionally, in separate experiments, MMECs treated with metformin alone (2 mM) or a combination of 2DG (5 mM) and metformin (2 mM) were also exposed to dorsomorphin/compound C (2 μM), a pharmacological inhibitor of AMPK, for the initial 8 h OR the final 8 h of the 48 h treatment protocol. Prolonged treatment (48 h) with compound C was not performed since compound C reportedly inhibits the activity of several other kinases with a higher potency than its inhibition of AMPK [78,79].

It must be noted that ‘normal/untreated/non-treated controls’, when mentioned in the manuscript, are time- and condition-matched sham controls in which the normal basal media contain 11 mM glucose.

### 4.4. Cell Proliferation Assay (MTS Assay)

Briefly, 1 × 10^4^ cells were plated in a 96-well plate and, on the following day, the cells were treated (see Section 4.3 on ‘cell treatments’) for 48 h, in triplicates, and the rate of cell proliferation was analyzed using the CellTiter 96^®^ AQ_ueous_ One Solution Cell Proliferation Assay kit (Cat # G3582, Promega Corporation, Madison, WI, USA) as per kit protocol, as previously described [90]. The absorbance (quantity of formazan product) was finally read using a 96-well ‘EnVision 2104 multilabel’ plate reader (PerkinElmer, Inc. Waltham, MA, USA) at 490 nm and is directly proportional to the number of living cells in culture. Background subtraction was performed using the values obtained from the wells containing just media and the reagent (without cells) [90].

### 4.5. Glycolysis Assay

The rate of glycolysis was analysed as per kit protocol using Glycolysis Cell-Based Assay Kit (Cat # 600450, Cayman Chemical, Ann Arbor, MI, USA). Briefly, 1 × 10^4^ cells were plated in a 96-well plate (plate A) and on the following day, the cells were treated for 48 h, in triplicates, as described (see Section 4.3 on ‘cell treatments’). Following the treatment period, the plate ‘A’ was centrifuged at 1000 rpm for 5 min. In a fresh pre-labelled 96-well plate (plate B) 90 μL of assay buffer was loaded into each well. A quantity of 5 μL of supernatant from each well of plate ‘A’ was transferred to corresponding wells on plate ‘B’ containing the 90 μL of assay buffer. A quantity of 10 μL of lactate standards was also loaded similarly (in duplicates). A quantity of 100 μL of reaction solution was added to each well of plate ‘B’ and the plate was incubated on an orbital shaker for 30 min at room temperature, after which the absorbance was read at 490 nm. The assay enabled colorimetric detection of L-lactate (the end product of glycolysis produced and secreted by cultured cells). The reaction between NAD^+^ and lactate was catalyzed by the lactate dehydrogenase enzyme, thus yielding pyruvate and NADH. The coloured formazan compound, formed by the NADH-based reduction of the tetrazolium salt, read at 490 nm (on a multiplate reader, CLARIOstar^®^ (BMG-LABTECH, Cary, NC, USA), proportional to the lactate concentration in the culture media thus provided an indirect measurement of glycolysis.

### 4.6. Cell Migration Assay

Cell migration was analysed as per kit protocol using the Radius™ 24-Well Cell Migration Assay (Cat # CBA-125, Cell Biolabs, Inc., San Diego, CA, USA). Briefly, 500 µL of Radius™ Gel Pretreatment Solution was added to the wells of the 24-well plate provided and was incubated for 20 min at room temperature. The pre-treatment solution was removed and Radius™ Wash Solution (500 µL) was added into each well and maintained at RT for 30 min. The Radius™ Wash Solution was aspirated and 1.5 × 10^5^ cells were then loaded into each well and the cells were allowed to form a monolayer and attain 90% confluency. The media was removed on the following day, and the wells were washed three times using 0.5 mL of fresh media, followed by aspiration of the media, addition of 1X Radius™ Gel Removal Solution (500 µL) to each well and incubation in a cell culture incubator at 37 °C for 30 min in order to facilitate complete gel removal. After removing the 1× Radius™ Gel Removal Solution, the wells were washed three times with 0.5 mL of fresh media. The cells were then treated in triplicates for each treatment condition as described (see Section 4.3 on ‘cell treatments’). Pre-migration images were captured with an inverted microscope after the addition of the treatment media (zero time; 0 h) and then images were captured at regular intervals of 12 h, 24 h, 36 h and 48 h. The wound healing observed here will be an outcome of cell migration and proliferation.

In order to assess the effect of our treatment on cell migration alone, thymidine (2 mM) was used to block cell proliferation and cell migration assay was performed as per kit protocol using the Cytoselect™ 24-Well Cell Migration Assay (Cat # CBA-120, Cell Biolabs, Inc., San Diego, CA, USA). Briefly, 1.5 × 10^5^ cells were loaded into each well and the cells were allowed to form a monolayer and attain 90% confluency. The insert (provided with the kit) in each well was then removed to generate a 0.9 mm ‘wound field’. The cells were then treated in triplicates for each treatment condition as described (see Section 4.3 on ‘cell treatments’) without thymidine. Alternatively, the treatment media for another set of cells in triplicates, for each treatment condition, contained 2 mM thymidine to block cell proliferation. Pre-migration images were captured with an inverted light microscope after the addition of the treatment media (zero time; 0 h) and then images were captured at intervals of 24 h and 48 h.

### 4.7. Tubulogenesis Assay

Tubulogenesis assay was performed as previously described [91]. MMECs were treated as described in Section 4.3 on ‘cell treatments’. In addition to the normal glucose levels (11 mM) in the treatment media, MMECs (p5–p10) were exposed to 2DG (5 mM) in the absence or presence of metformin (2 mM) for 24 h prior to tubulogenesis assay. Non-treated normal glucose-exposed cells in the absence or presence of metformin were considered as normal controls. These cells were then used for matrigel assay. In brief, 350 μL of ice cold Geltrex™ LDEV-free reduced growth factor basement membrane matrix (Cat # A1413202; Thermo-Fisher Scientific, Waltham, MA, USA) was coated on a 24-well cell culture plate to prepare a base for the tube formation assay. The gel was allowed to settle for 30 min in a 5% CO_2_ incubator at 37 °C. After 24 h in culture, the MMECs were trypsinized and harvested and seeded (20,000 cells/well) onto the matrigel in their respective treatment media/conditions. We intended to image the cells after 2 h, 4 h, 6 h, 12 h and 24 h (to match the 48 h time point for the other experiments) on a matrigel. However, during initial experiments, it was noted that the tube/network formation dissipated within 6 h after the cells were transferred onto the basement membrane matrix and hence the experiment was restricted to 4 h and the images of the cells were captured using a camera mounted on an inverted light microscope after 2 h and 4 h on a geltrex matrix. Although we did see a variation in the tubulogenic effect of the cells under the different treatments (Figure 7B), we did not quantify the data in terms of tube length or number of 3-point junctions as this did not match the 48 h time point of the other data presented in this study.

### 4.8. AMPK Gene Silencing

MMECs were grown on 60 mm plates in growth medium without antibiotics to attain 40–50% confluency at the time of transfection. AMPK gene silencing using AMPKα1/2 siRNA (Cat # sc45313; Santa Cruz Biotechnology, Inc., Dallas, TX, USA) was performed as previously described [16]. Scrambled siRNA (CsiRNA, Cat # sc37007; Santa Cruz Biotechnology, Inc., Dallas, TX, USA) was used as control.

### 4.9. Protein Isolation and Total Protein Estimation

After the completion of the treatment protocols, protein lysates were prepared and the protein concentrations of each sample were analyzed as previously described [16,49,90], followed by preparation of samples for SDS-PAGE and immuno-blotting.

### 4.10. Immunoblotting

SDS-Polyacrylamide Gel Electrophoresis (SDS-PAGE) followed by immuno-blotting was used to detect the levels of TSP1, phospho-VEGFR2 (Y1175), VEGFR2, phospho-AMPK (T172), AMPK, phospho-cyclin B1 (S147), cyclin B1, cyclin D1, cyclin D2, phospho-Akt (S473), Akt, phospho-mTOR (S2448), mTOR, phospho-raptor (S792), raptor, phospho-4E-BP1 (T37/46), 4E-BP1, phospho-S6 ribosomal protein (S235/236), phospho-S6 ribosomal protein (S240/244), S6 ribosomal protein, caspase-3, cleaved caspase-3 and OCTs (1, 2 and 3) as previously described [16,49]. The respective total protein form was detected in the same blot after stripping, when probing for phospho-proteins [16]. We probed randomly for two loading controls, β-actin and β-tubulin, for Western blotting experiments to avoid over reliance on one single loading control. We have used the dual-colour protein standards (Cat # 161–0374, Bio-Rad, Inc., Hercules, CA, USA) that has the 10, 15, 20, 25, 37, 50, 75, 100, 150 and 250 kDa protein bands as our molecule weight marker. The protein bands after Western blotting were detected, imaged and the densities of bands obtained were then quantified as previously described [16]. All the original raw blots, ECL images and the image overlays indicating the proteins are provided in the Supplementary Material.

### 4.11. Immunofluorescence Staining for CD36-TSP1 Co-Localization Studies

MMECs grown on coverslips in 24-well plates and subjected to various treatments (as described in Section 4.3 on ‘cell treatments’) were used for immunofluorescence staining and detection of CD36 and TSP1 and their co-localization, as per the protocol previously described [16]. TSP1 expression was detected using the primary anti-TSP1 antibody (raised in rabbit, Cat # ab85762; Abcam Inc., Cambridge, MA, USA; 1:100 in 5% NHS in PBS) antibody while primary anti-CD36 antibody (raised in mouse, Cat # sc-7309; Santa Cruz Biotechnology, Inc., Dallas, TX, USA; 1:100 in 5% NHS in PBS) was used to detect CD36 expression. The cells were visualized, and fluorescence images were captured using the LSM880, confocal laser scanning microscope (Zeiss, Inc., Oberkochen, Germany).

### 4.12. Statistical Analysis

The statistical software GraphPad Prism 7.0 (GraphPad Software, Inc., San Diego, CA, USA) was used to analyze the data obtained from the experiments and was represented as mean ± SEM from 4 to 5 experiments. One-way analysis of variance (ANOVA) followed by post-hoc comparisons between groups (by Tukey’s multiple comparison tests) were carried out to test for statistical significance (*p* < 0.05).

## 5. Conclusions

Our data demonstrates that using metformin in combination with 2DG results in a robust upregulation of anti-angiogenic TSP1. Additionally, using a combination treatment of metformin and 2DG significantly inhibited the mTOR pathway, decreased the levels of cell-cycle-related proteins and inhibited cell proliferation and migration and thus could prove to be a potential anti-proliferative and anti-angiogenic therapeutic strategy in the treatment of several cancers.

## Supplementary Materials

The following are available online at https://www.mdpi.com/2072-6694/11/11/1737/s1, Figure S1. Original unedited blot, ECL images and image overlays indicating TSP1 and β-actin for representative Western blots used in Figure 1A of the manuscript, Figure S2. Original unedited blot, ECL images and image overlays indicating TSP1 and β-actin for representative Western blots used in Figure 1B of the manuscript, Figure S3. Original unedited blot, ECL images and image overlays indicating TSP1 and β-actin for representative Western blots used in Figure 1C of the manuscript, Figure S4. Original unedited blot, ECL images and image overlays indicating TSP1, pVEGFR2 (Y1175), VEGFR2 and β-actin for representative Western blots used in Figure 2B of the manuscript, Figure S5. Original unedited blot, ECL images and image overlays indicating TSP1 for representative Western blots used in Figure 2C of the manuscript, Figure S6. Original unedited blot, ECL images and image overlays indicating β-tubulin and β-actin for representative Western blots used in Figure 2C of the manuscript, Figure S7. Original unedited blot, ECL images and image overlays indicating pVEGFR2 (Y1175), VEGFR2 and β-actin for representative Western blots used in Figure 2F of the manuscript, Figure S8. Original unedited blot, ECL images and image overlays indicating pAkt (S473), Akt and β-actin for representative Western blots used in Figure 3A of the manuscript, Figure S9. Original unedited blot, ECL images and image overlays indicating pmTOR (S2448), mTOR and β-actin for representative Western blots used in Figure 3B of the manuscript, Figure S10. Original unedited blot, ECL images and image overlays indicating pRap (S792) and Raptor for representative Western blots used in Figure 3B of the manuscript, Figure S11. Original unedited blot, ECL images and image overlays indicating p4E-BP1 (T37/46), and p4E-BP1 for representative Western blots used in Figure 3C of the manuscript, Figure S12. Original unedited blot, ECL images and image overlays indicating pS6 (S235/236), pS6 (S240/244), S6 ribosomal protein and β-actin for representative Western blots used in Figure 3C of the manuscript, Figure S13. Original unedited blot, ECL images and image overlays indicating pCycB1 (S147) and CycB1 for representative Western blots used in Figure 4A of the manuscript, Figure S14. Original unedited blot, ECL images and image overlays indicating CycD1, CycD2 and β-actin for representative Western blots used in Figure 4A of the manuscript, Figure S15. Original unedited blot, ECL images and image overlays indicating pAMPKα (T172), AMPK and β-actin for representative Western blots used in Figure 5A of the manuscript, Figure S16. Original unedited blot, ECL images and image overlays indicating TSP1 and β-actin for representative Western blots used in Figure 5D of the manuscript, Figure S17. Original unedited blot, ECL images and image overlays indicating TSP1 and β-actin for representative Western blots used in Figure 5F of the manuscript, Figure S18. Original unedited blot, ECL images and image overlays indicating TSP1, and β-tubulin and β-actin for representative Western blots used in Figure 5G of the manuscript, Figure S19. Original unedited blot, ECL images and image overlays indicating Caspase-3 and Cleaved Caspase-3 for representative Western blots used in Figure 7A of the manuscript, Figure S20. Original unedited blot, ECL images and image overlays indicating β-tubulin and β-actin for representative Western blots used in Figure 7A of the manuscript, Figure S21. Original unedited blot, ECL images and image overlays indicating Cleaved Caspase-3, β-tubulin and β-actin for representative Western blots used in Figure 7B of the manuscript, Figure S22. Original unedited blot, ECL images and image overlays indicating OCT1 for representative Western blots used in Figure 10A of the manuscript, Figure S23. Original unedited blot, ECL images and image overlays indicating β-tubulin and β-actin for representative Western blots used in Figure 10A of the manuscript, Figure S24. Original unedited blot, ECL images and image overlays indicating OCT2 and OCT3 for representative Western blots used in Figure 10A of the manuscript.

## Abbreviations

μΜ	Micromolar
μm	Micrometre
2DG	2-deoxyglucose
4E-BP1	Eukaryotic translation initiation factor 4E-binding protein 1
Akt	Protein kinase B
AMPK	AMP activated protein kinase
CD36	Platelet glycoprotein IV/scavenger receptor class B member 3
CycB1	Cyclin B1
CycD1	Cyclin D1
CycD2	Cyclin D2
ECs	Endothelial cells
h	Hours
HER2	Human epidermal growth factor receptor 2
HIF1α	Hypoxia inducible factor-1 alpha
HUVECs	Human umbilical vein endothelial cells
MATE1/2	Multidrug and toxin extrusion protein 1 and 2
min	Minutes
mM	Millimolar
MMECs	Mouse microvascular endothelial cells
mTOR	Mammalian target of rapamycin
nm	Nanometre
PMAT	Plasma membrane monoamine trasporter
Rap	Raptor
SHP1	Src-homology 2-containing tyrosine phosphatase-1
TECs	Tumor endothelial cells
TSP1	Thrombospondin 1
VEGF	Vascular endothelial growth factor
VEGFR2	Vascular endothelial growth factor receptor 2
