# Supplementary material for: Treatment with a Combination of Metformin and 2-Deoxyglucose Upregulates Thrombospondin-1 in Microvascular Endothelial Cells: Implications in Anti-Angiogenic Cancer Therapy
Source: Cancers (Basel). 2019 Nov 6;11(11):1737. doi: 10.3390/cancers11111737 (PMC6895998; doi:10.3390/cancers11111737)
Supplement: Supplementary file 1 [file cancers-11-01737-s001.pdf]

Supplementary materials: Treatment with a Combination of Metformin and 2-Deoxyglucose Upregulates Thrombospondin-1 in Microvascular Endothelial Cells: Implications in Anti-Angiogenic Cancer Therapy

Samson Mathews Samuel, Noothan Jyothi Satheesh, Suparna Ghosh, Dietrich Büsselberg, Yasser Majeed, Hong Ding and Chris R. Triggle

Description on Western blot imaging, use of markers and overlaying/superimposing

In the supplemental materials section, we have provided all the original raw blots, ECL images and the image overlays indicating the proteins we have probed for. We have *'marked'* the molecular weight markers on the Western blot images in the final figures of the manuscript.

The molecular weight marker that we have used in the current study is the dual colour protein standards (Cat # 161-0374, Biorad, Inc., Hercules, CA, USA) having the 10, 15, 20, 25, 37, 50, 75, 100, 150 and 250 kDa protein markers. The date of SDS-PAGE and Western blotting, sample ID (with date of isloation), date of protein estimation, sequence of loading and amount of protein loaded was recorded and saved for future reference. The marker was run in the first lane alongside our protein samples in every SDS-PAGE/Western blot performed. The standards used, however cannot be imaged with enhanced chemiluminescent (ECL) solution and do not appear on the final image, although efficiently transferred from the gel to the nitrocellulose (NC) membrane. We have used the Geliance P600 gel documentation system (PerkinElmer, Inc., Waltham, MA, USA). Therefore, we capture the raw colorimetric image of NC membrane/blot that has the transferred protein markers and then add ECL to probe for our protein of interest. After developing the blots, the ECL image (35-40% transparency) was superimposed onto the colorimetric image (containing the marker bands) to identify our protein of interest.

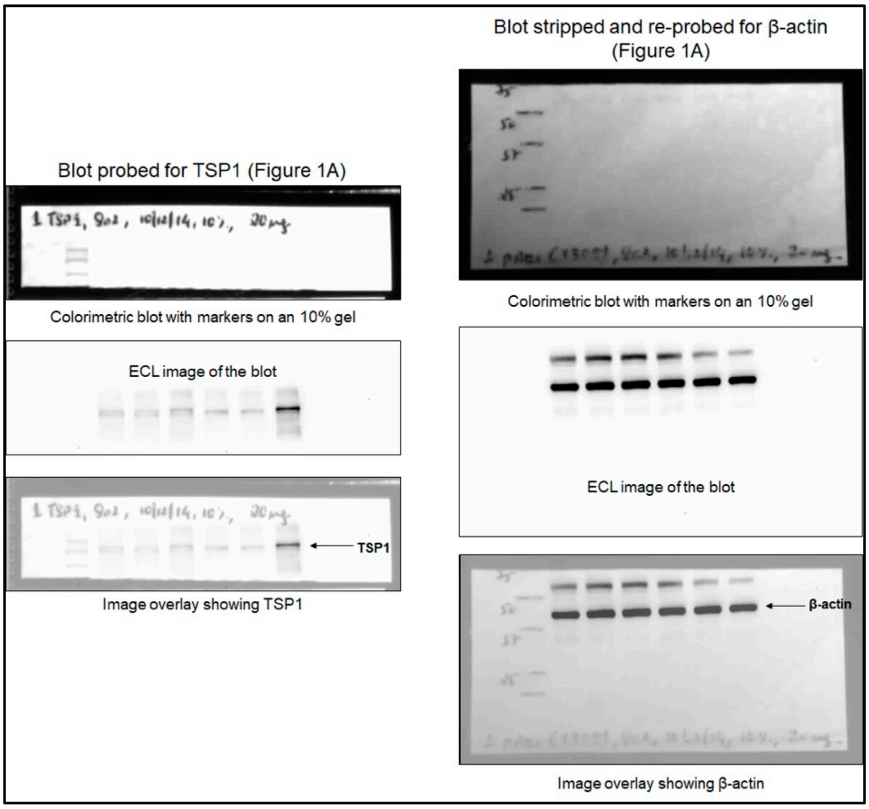

Figure S1. Original unedited blot, ECL images and image overlays indicating TSP1 and β-actin for representative Western blots used in Figure 1A of the manuscript.

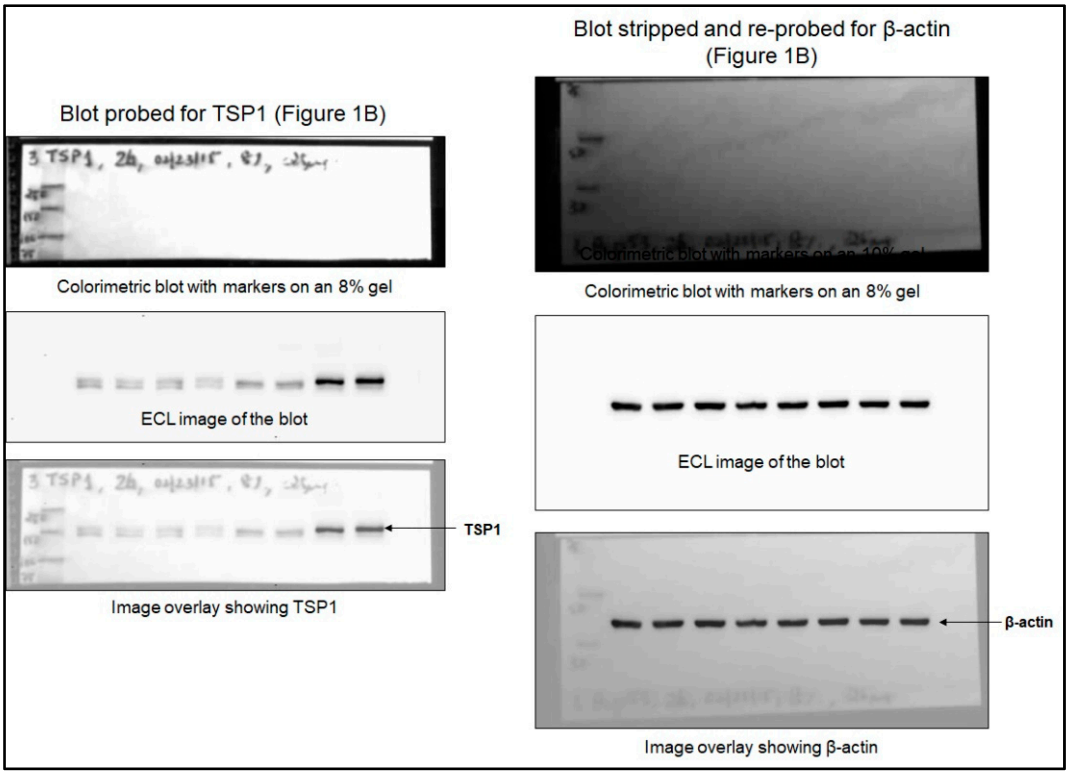

Figure S2. Original unedited blot, ECL images and image overlays indicating TSP1 and β-actin for representative Western blots used in Figure 1B of the manuscript.

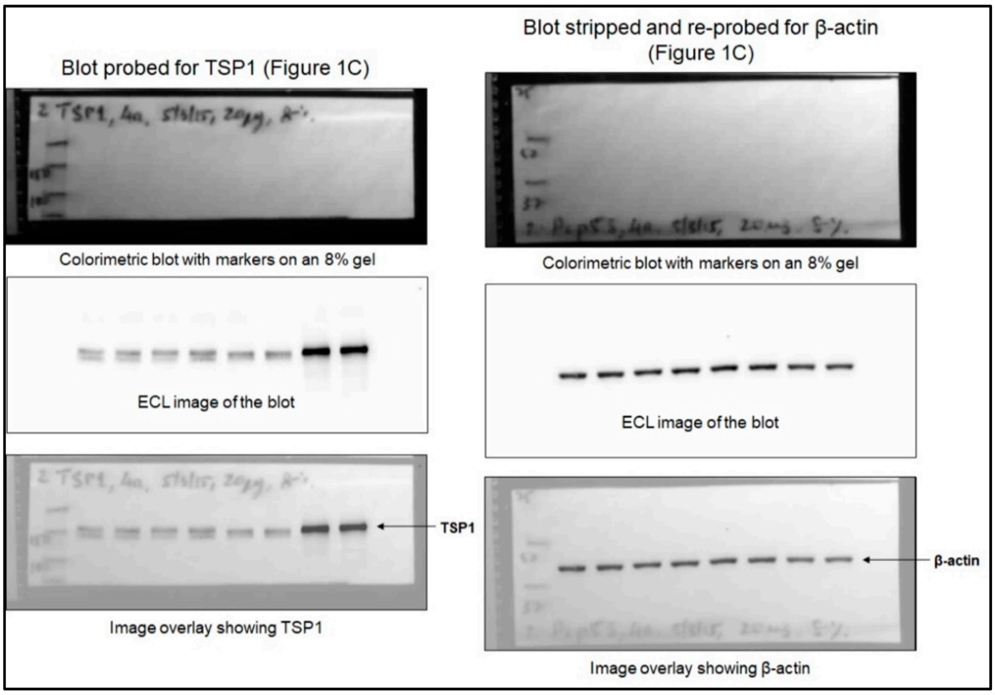

Figure S3. Original unedited blot, ECL images and image overlays indicating TSP1 and β-actin for representative Western blots used in Figure 1C of the manuscript.

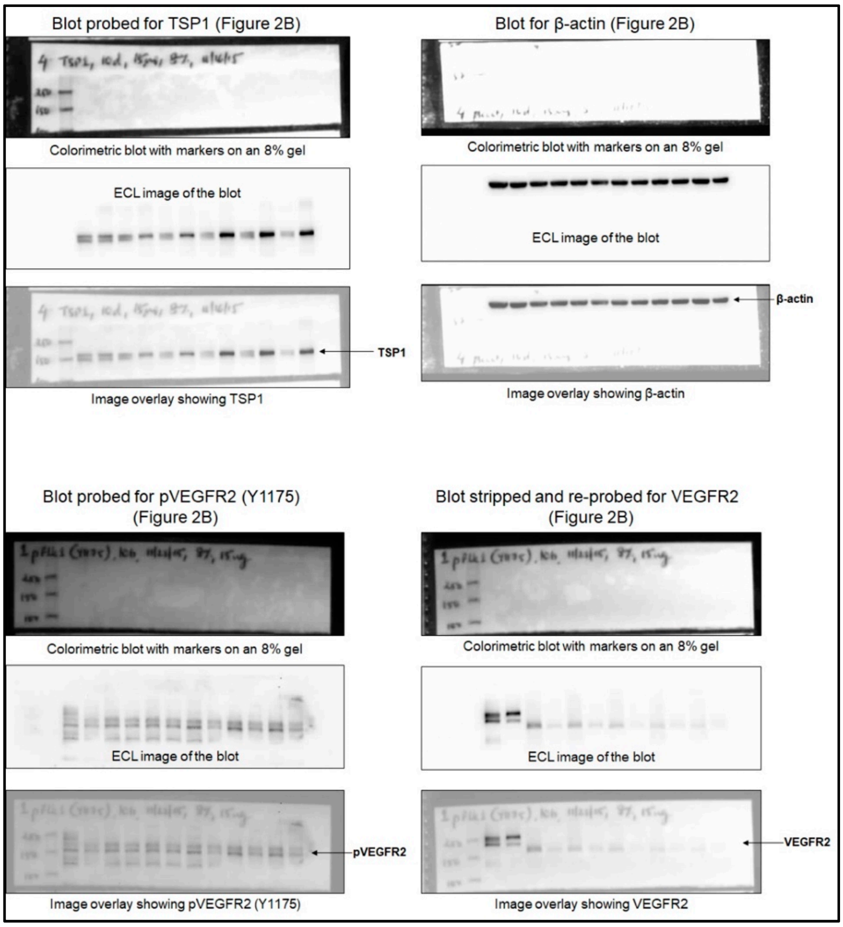

Figure S4. Original unedited blot, ECL images and image overlays indicating TSP1, pVEGFR2 (Y1175), VEGFR2 and β-actin for representative Western blots used in Figure 2B of the manuscript.

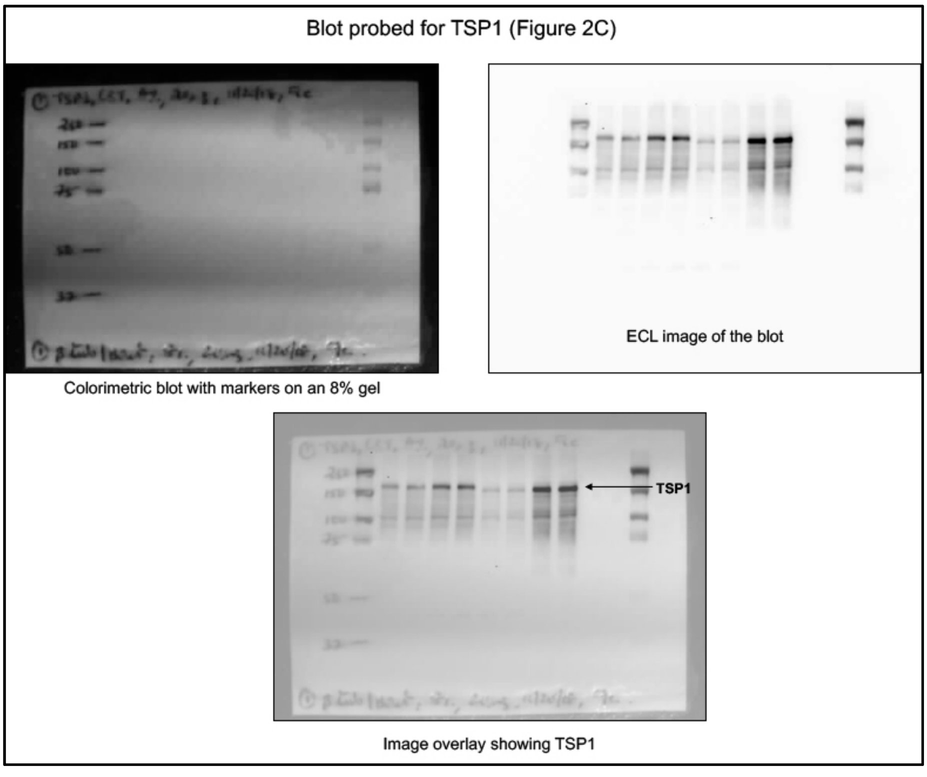

Figure S5. Original unedited blot, ECL images and image overlays indicating TSP1 for representative Western blots used in Figure 2C of the manuscript.

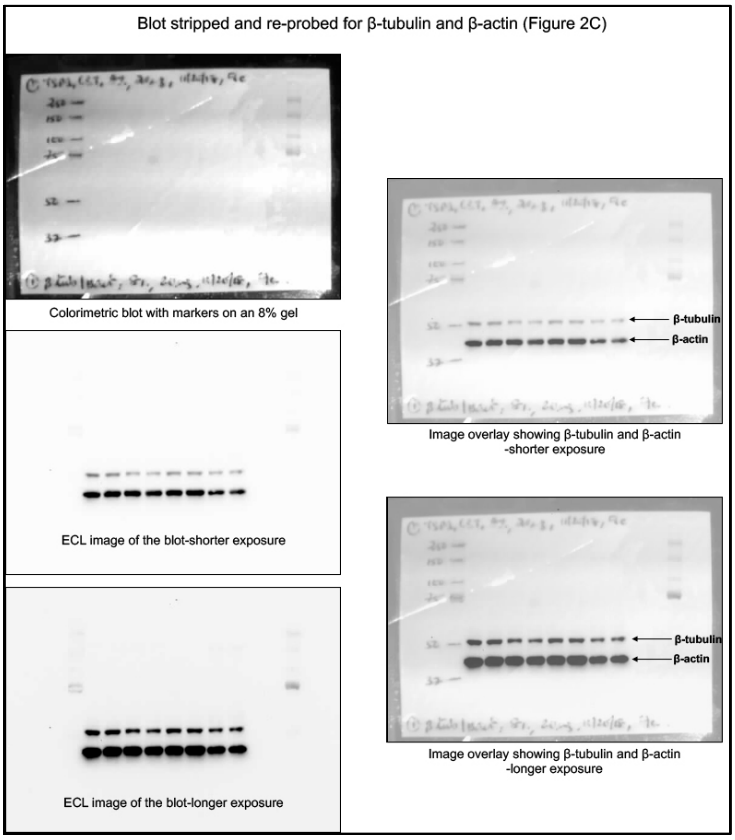

Figure S6. Original unedited blot, ECL images and image overlays indicating β-tubulin and β-actin for representative Western blots used in Figure 2C of the manuscript.

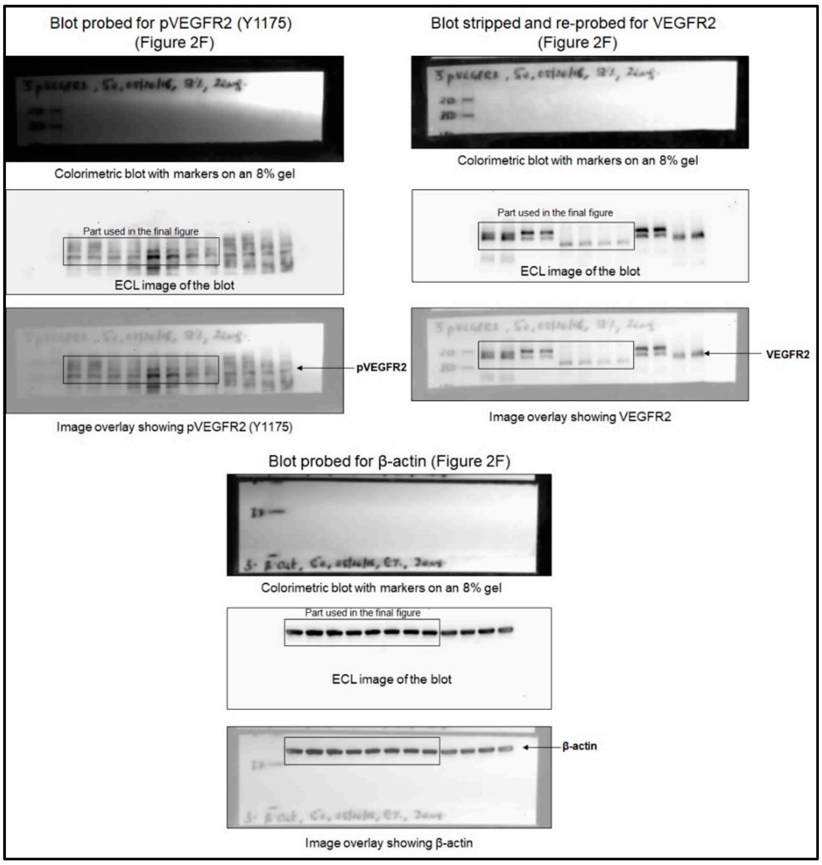

Figure S7. Original unedited blot, ECL images and image overlays indicating pVEGFR2 (Y1175), VEGFR2 and β-actin for representative Western blots used in Figure 2F of the manuscript.

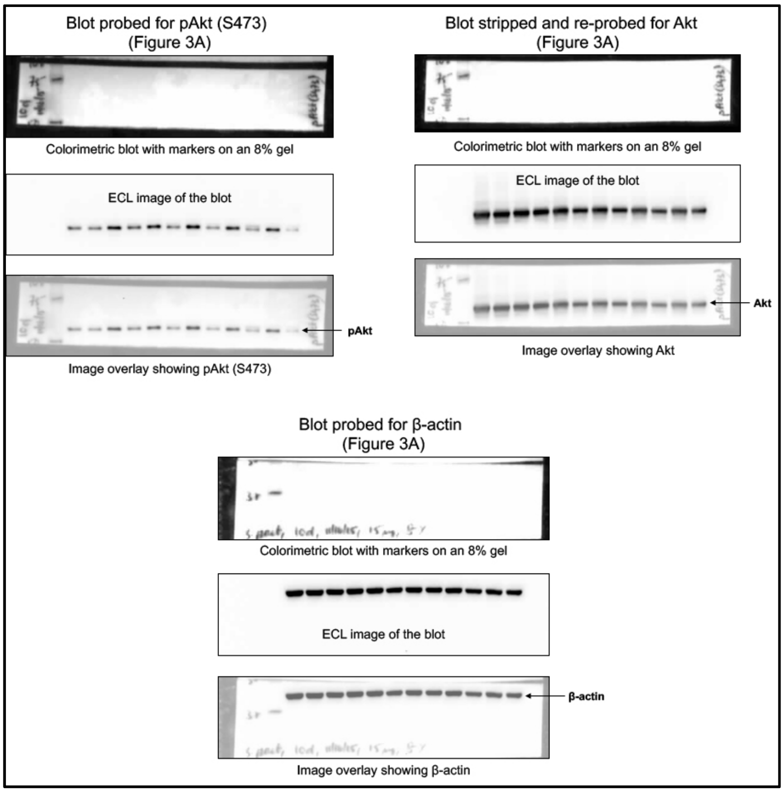

Figure S8. Original unedited blot, ECL images and image overlays indicating pAkt (S473), Akt and β-actin for representative Western blots used in Figure 3A of the manuscript.

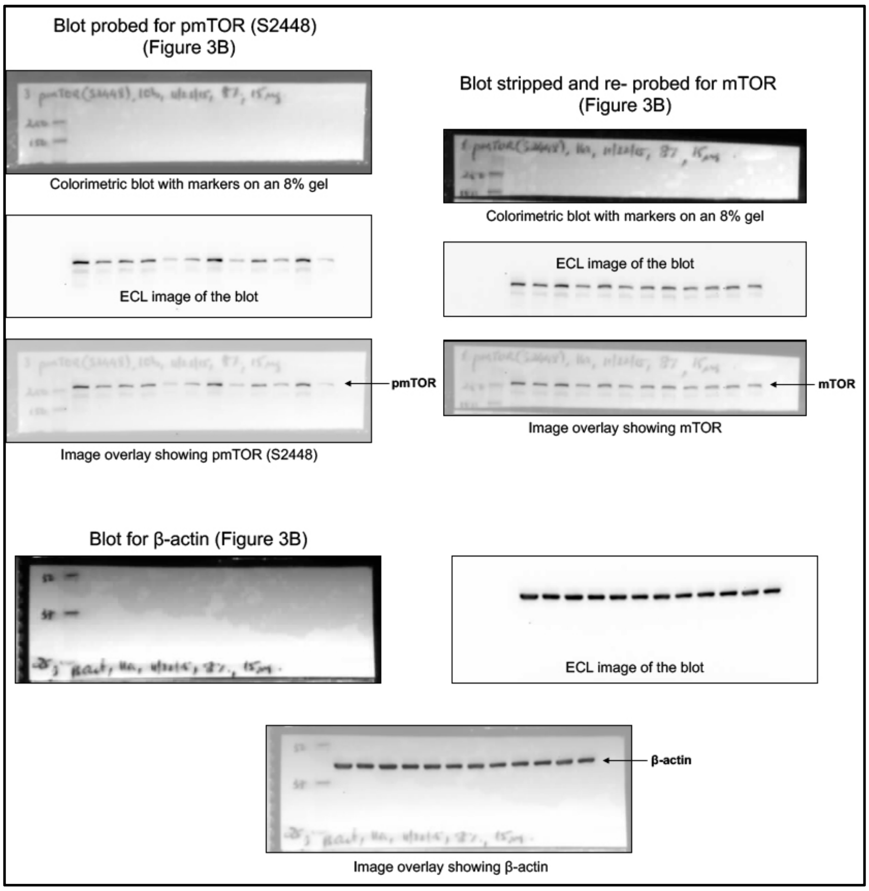

Figure S9. Original unedited blot, ECL images and image overlays indicating pmTOR (S2448), mTOR and β-actin for representative Western blots used in Figure 3B of the manuscript.

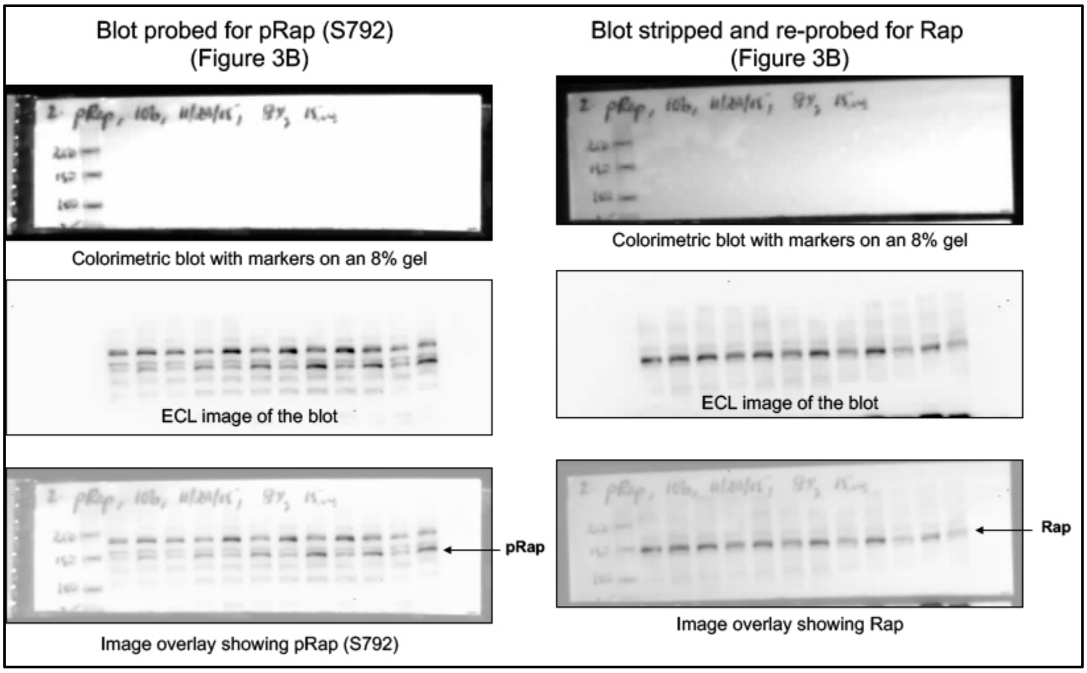

Figure S10. Original unedited blot, ECL images and image overlays indicating pRap (S792) and Raptor for representative Western blots used in Figure 3B of the manuscript.

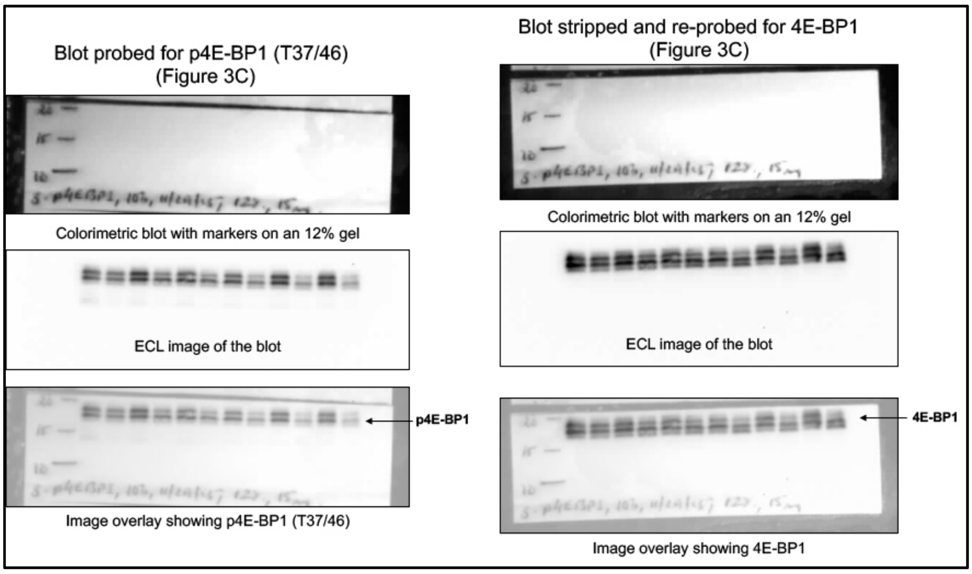

Figure S11. Original unedited blot, ECL images and image overlays indicating p4E-BP1 (T37/46), and p4E-BP1 for representative Western blots used in Figure 3C of the manuscript.

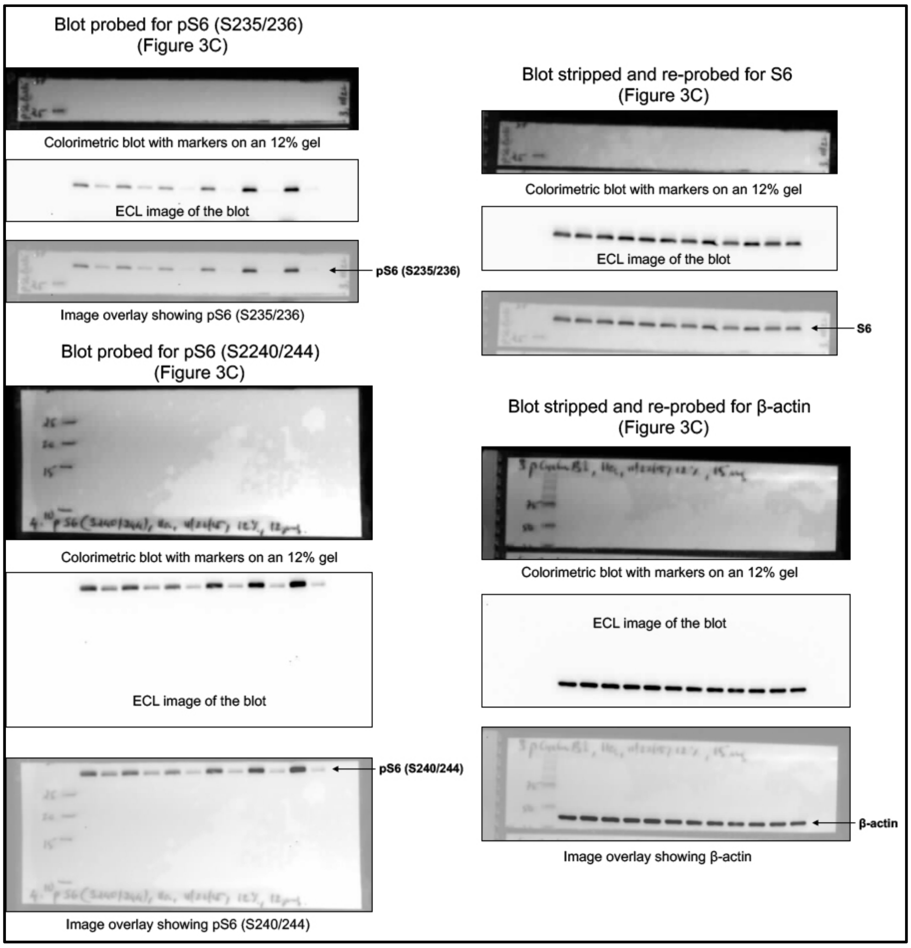

Figure S12. Original unedited blot, ECL images and image overlays indicating pS6 (S235/236), pS6 (S240/244), S6 ribosomal protein and β-actin for representative Western blots used in Figure 3C of the manuscript.

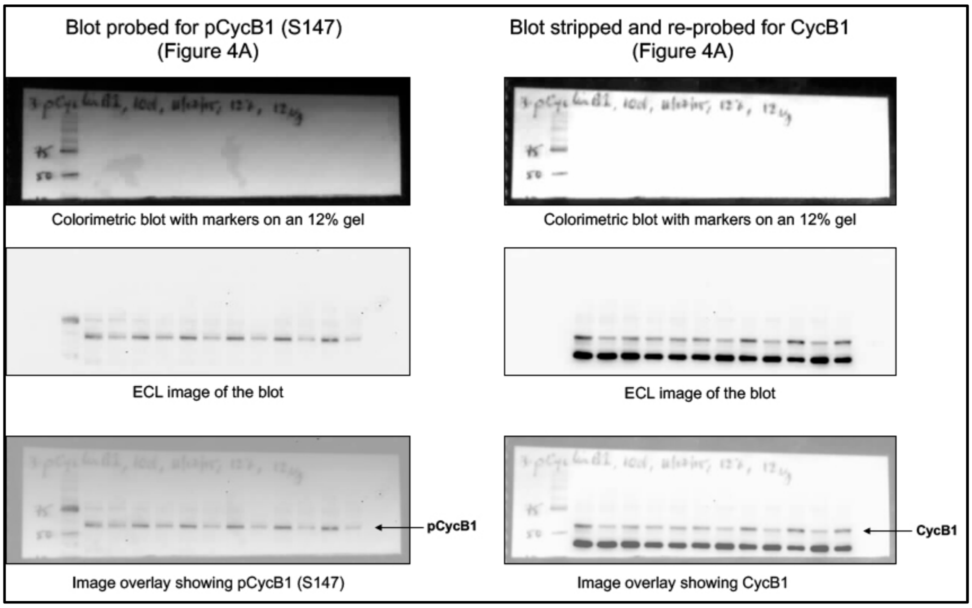

Figure S13. Original unedited blot, ECL images and image overlays indicating pCycB1 (S147) and CycB1 for representative Western blots used in Figure 4A of the manuscript

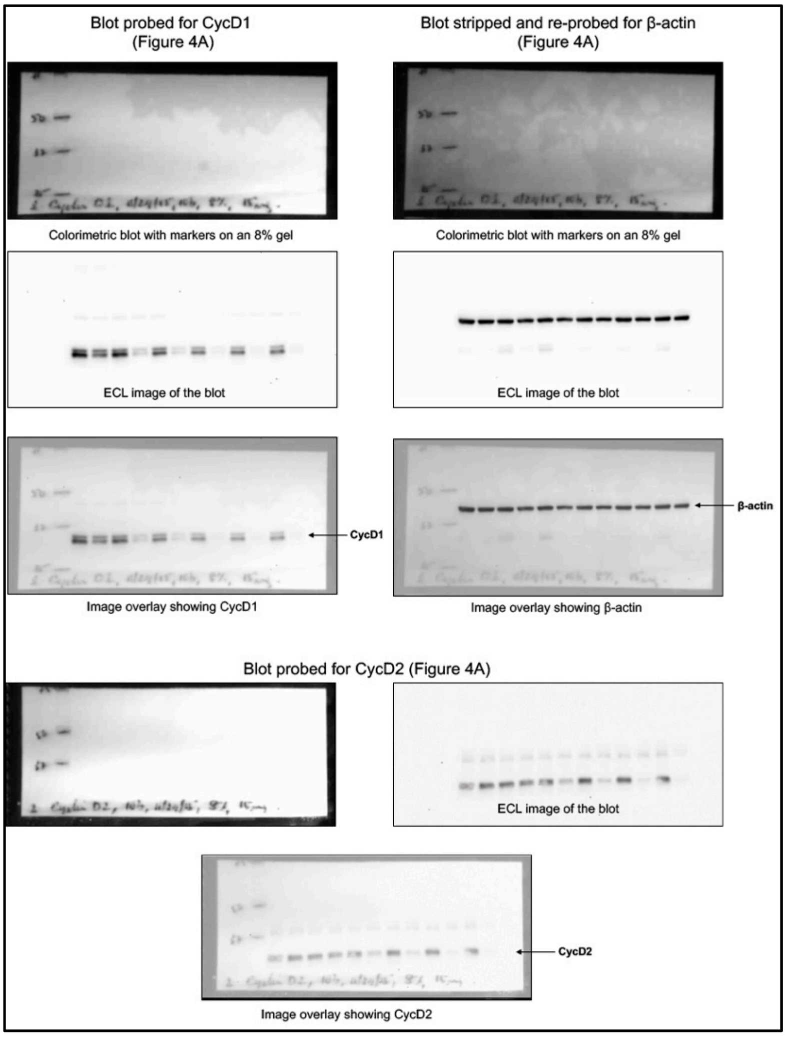

Figure S14. Original unedited blot, ECL images and image overlays indicating CycD1, CycD2 and β-actin for representative Western blots used in Figure 4A of the manuscript.

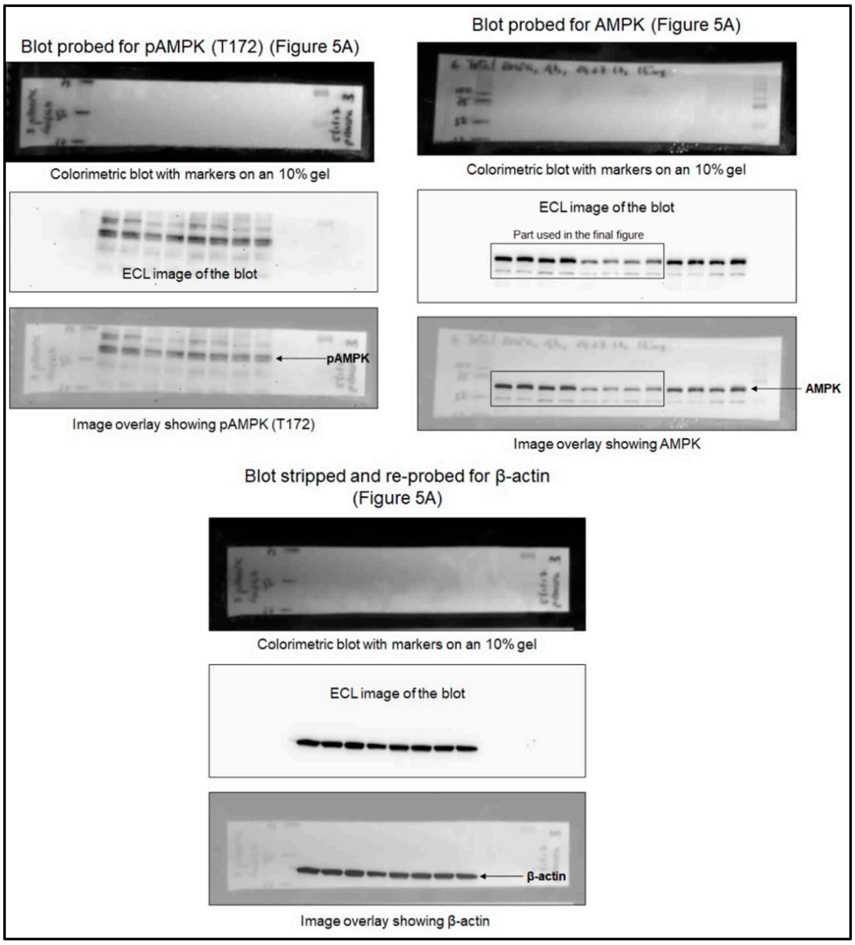

Figure S15. Original unedited blot, ECL images and image overlays indicating pAMPK (T172), AMPK and β-actin for representative Western blots used in Figure 5A of the manuscript.

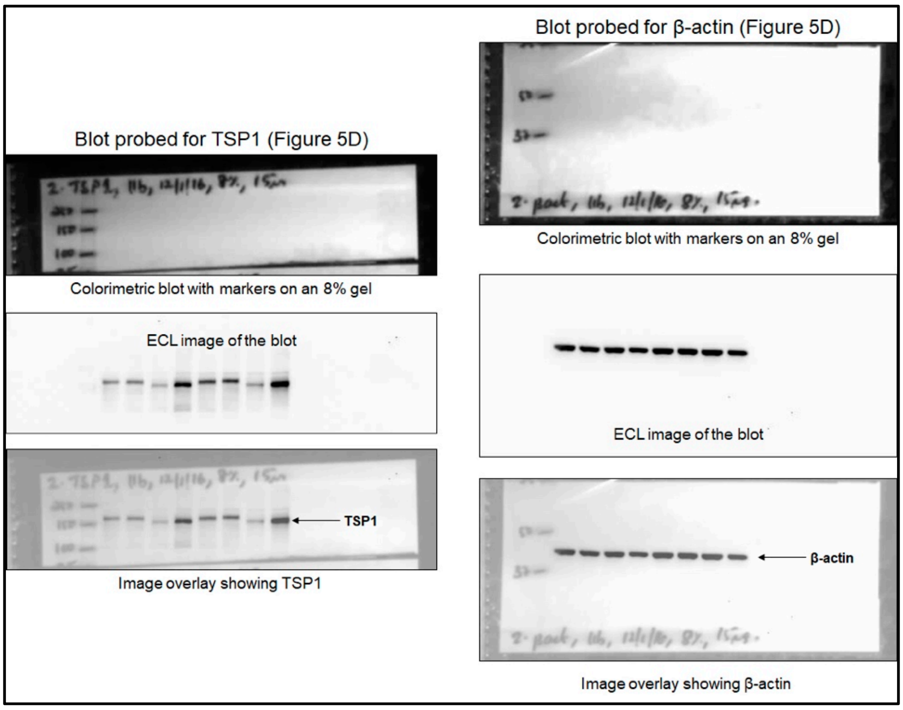

Figure S16. Original unedited blot, ECL images and image overlays indicating TSP1 and β-actin for representative Western blots used in Figure 5D of the manuscript.

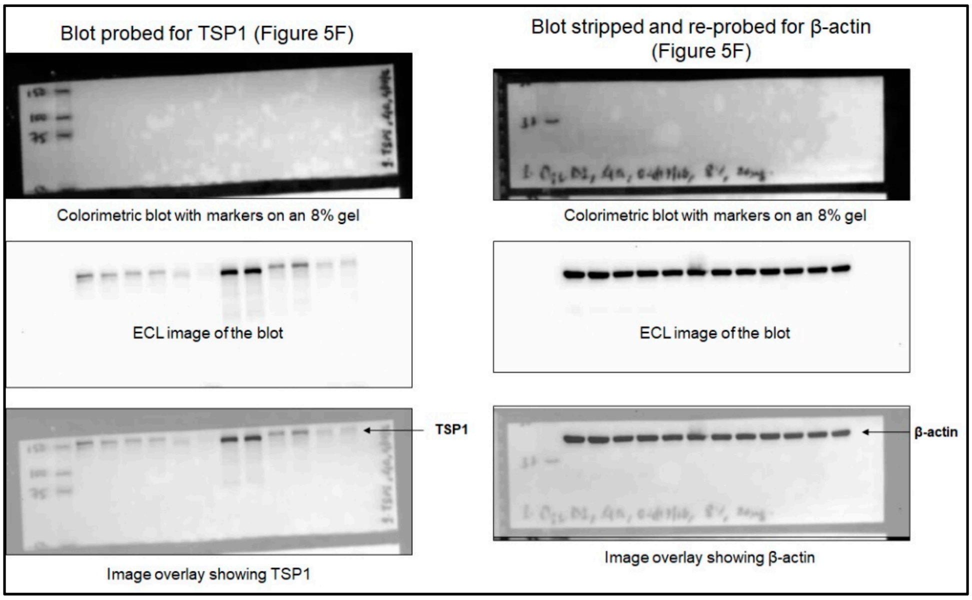

Figure S17. Original unedited blot, ECL images and image overlays indicating TSP1 and β-actin for representative Western blots used in Figure 5F of the manuscript.

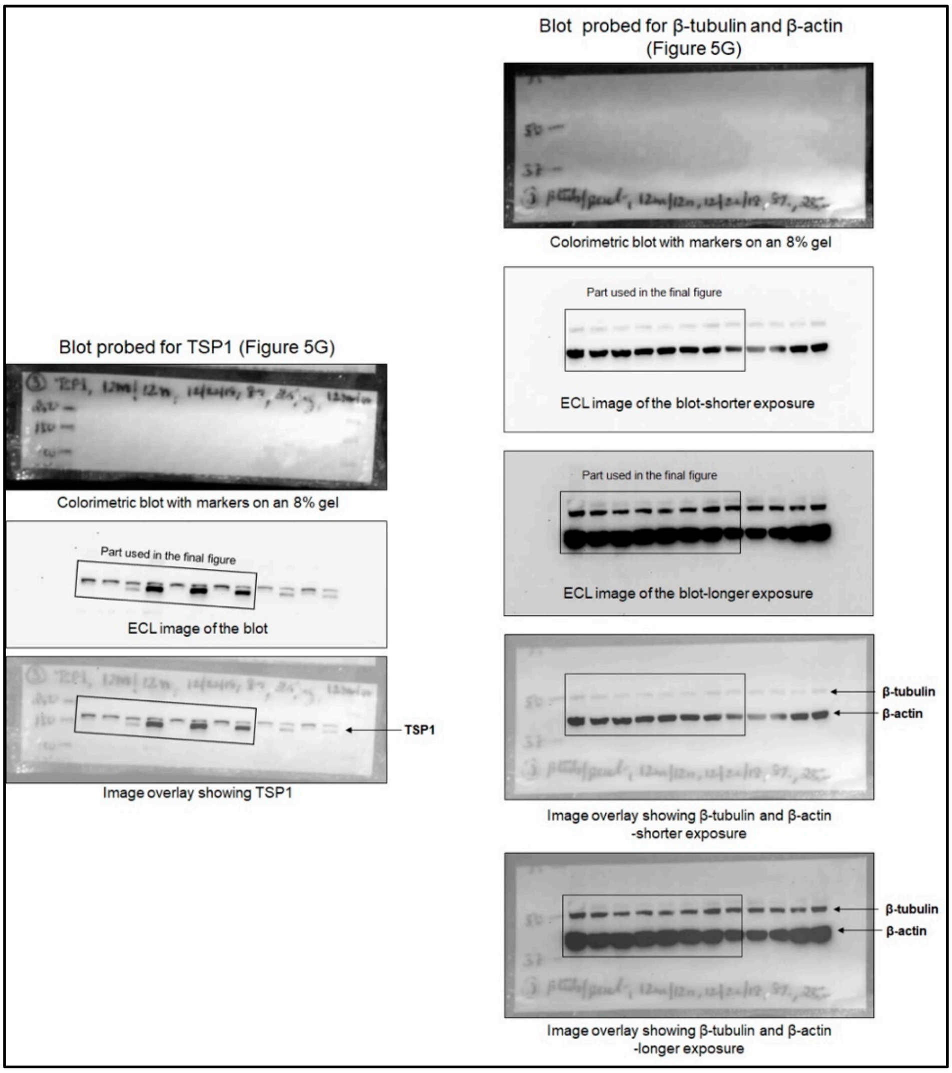

Figure S18. Original unedited blot, ECL images and image overlays indicating TSP1, and β-tubulin and β-actin for representative Western blots used in Figure 5G of the manuscript.

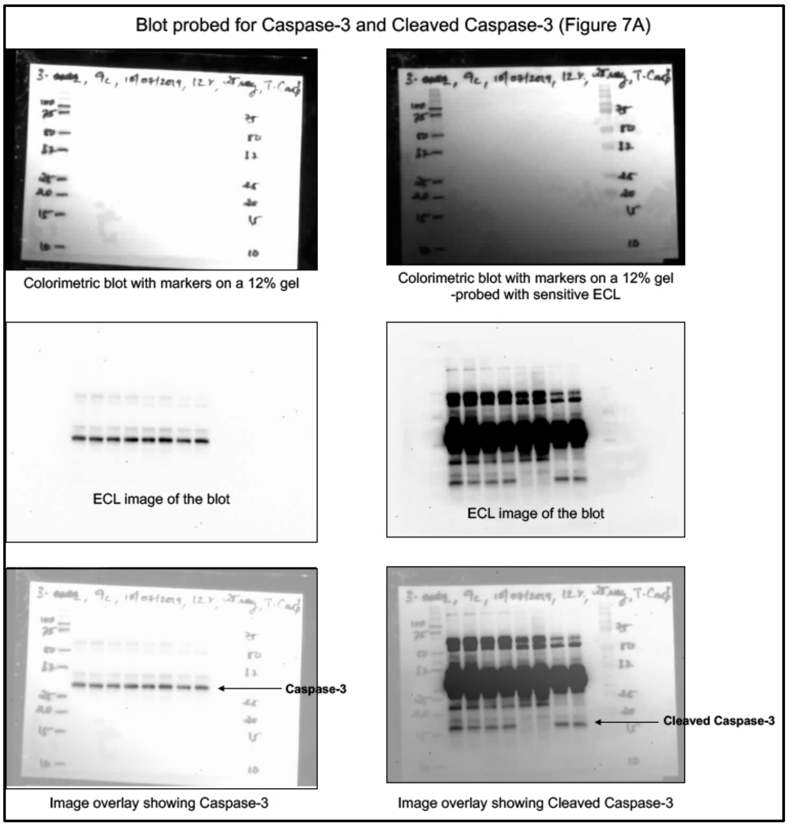

Figure S19. Original unedited blot, ECL images and image overlays indicating Caspase-3 and Cleaved Caspase-3 for representative Western blots used in Figure 7A of the manuscript.

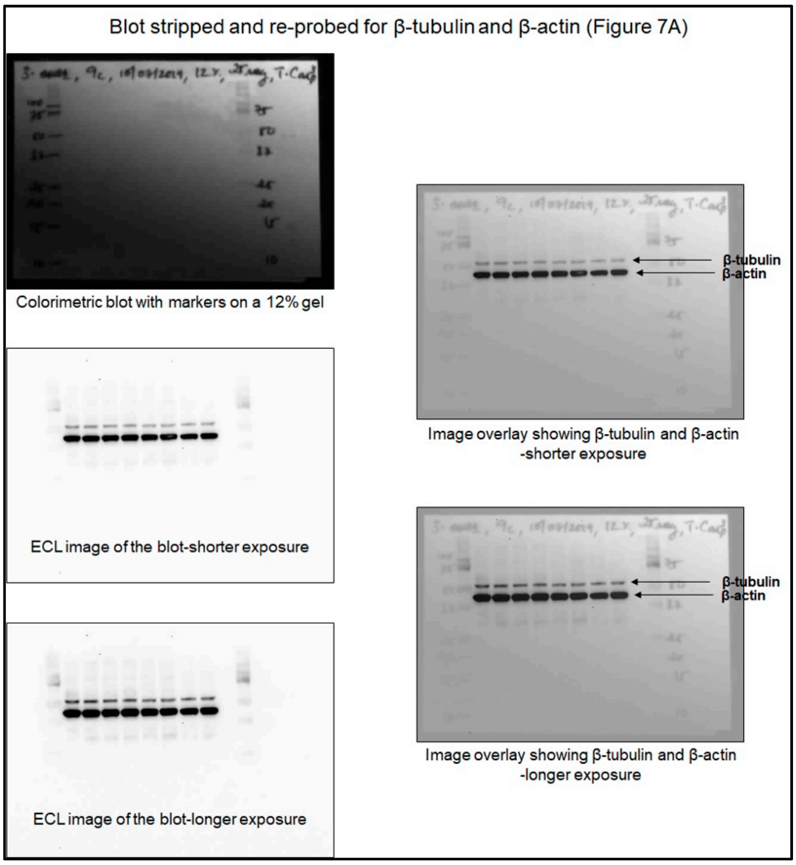

Figure S20. Original unedited blot, ECL images and image overlays indicating β-tubulin and β-actin for representative Western blots used in Figure 7A of the manuscript.

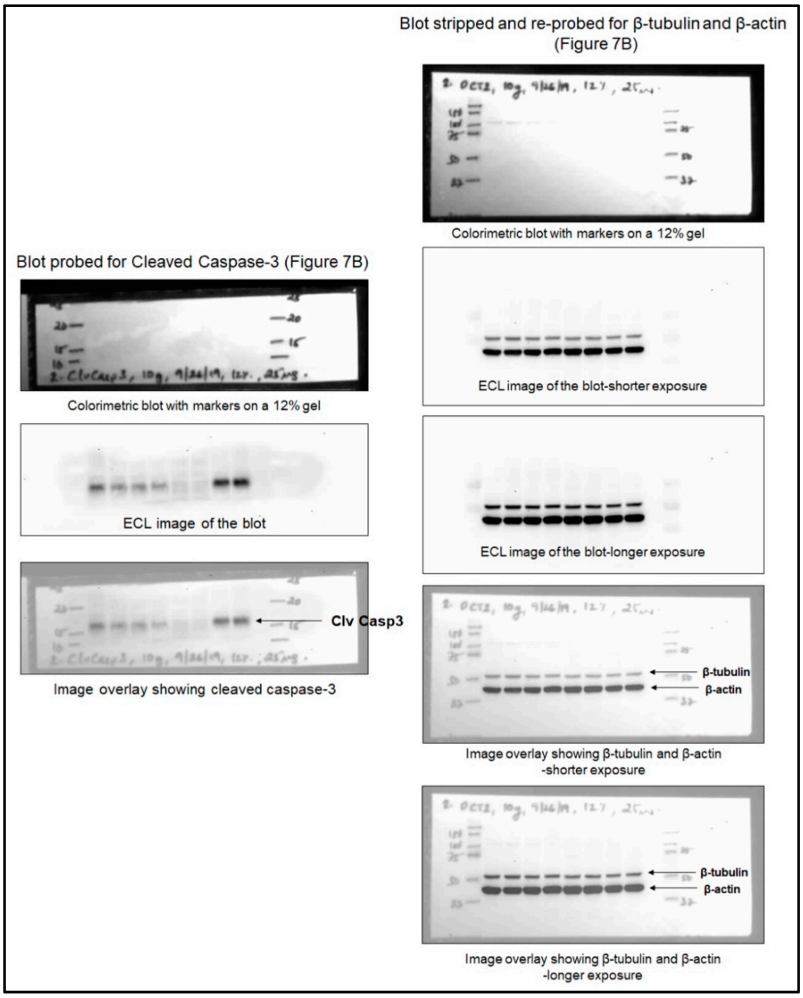

Figure S21. Original unedited blot, ECL images and image overlays indicating Cleaved Caspase-3, β-tubulin and β-actin for representative Western blots used in Figure 7B of the manuscript.

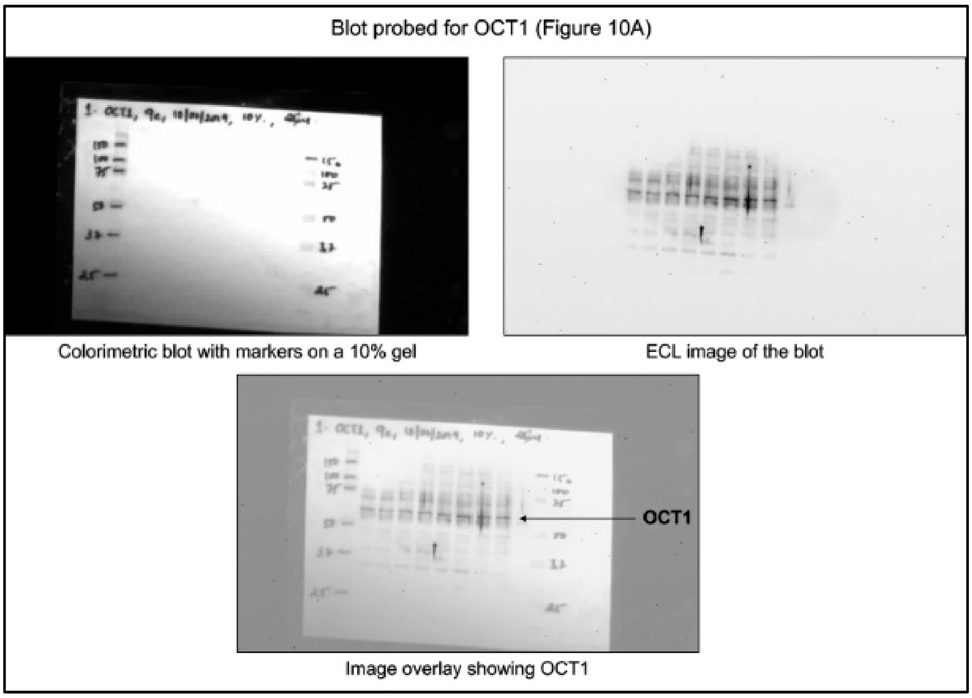

Figure S22. Original unedited blot, ECL images and image overlays indicating OCT1 for representative Western blots used in Figure 10A of the manuscript.

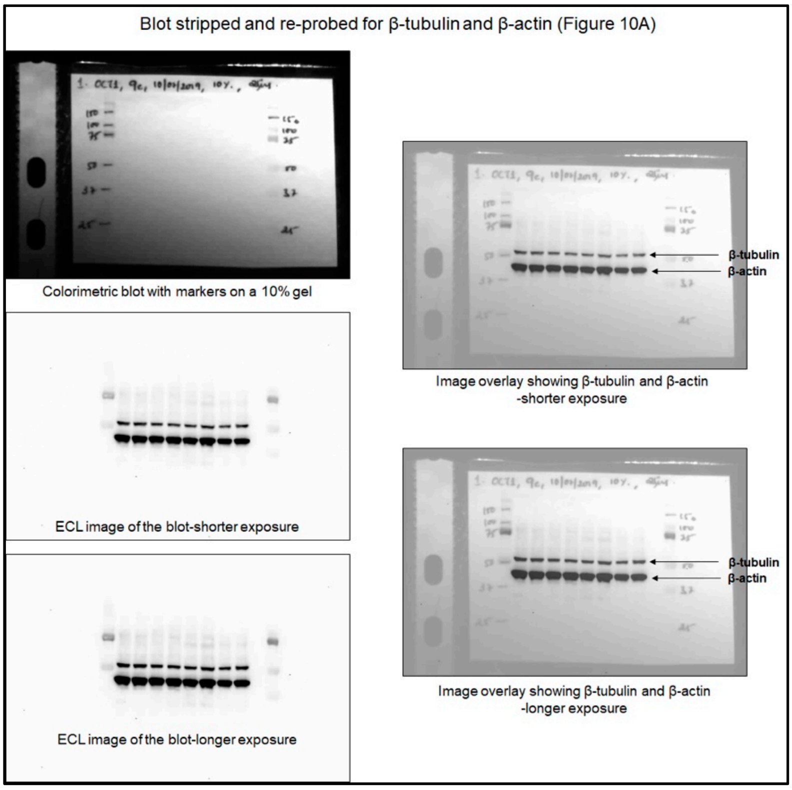

Figure S23. Original unedited blot, ECL images and image overlays indicating β-tubulin and β-actin for representative Western blots used in Figure 10A of the manuscript

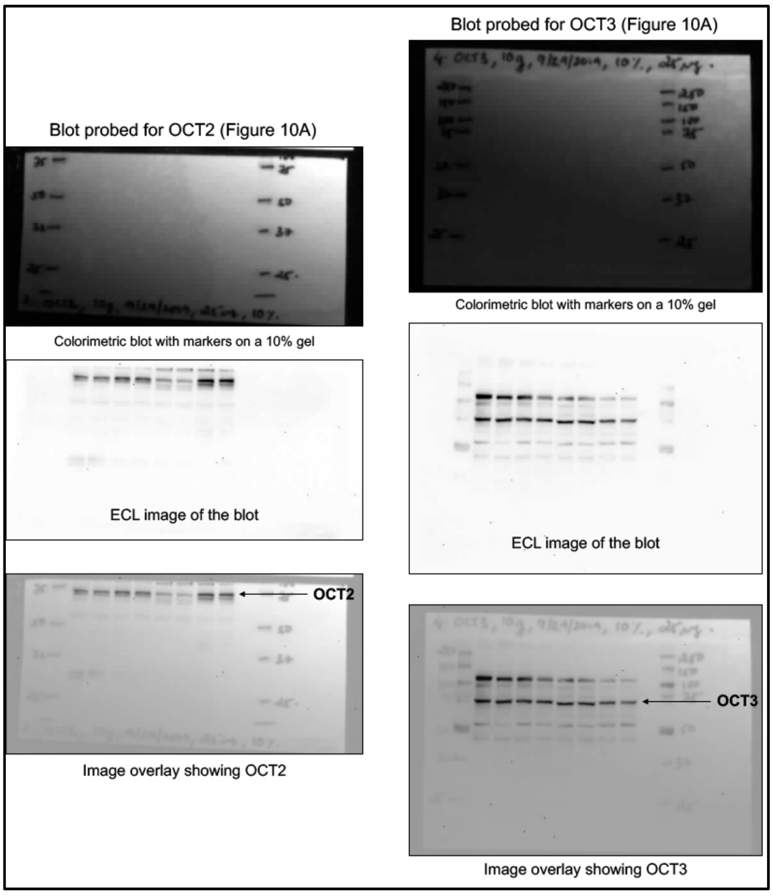

Figure S24. Original unedited blot, ECL images and image overlays indicating OCT2 and OCT3 for representative Western blots used in Figure 10A of the manuscript.
